# Both parents matter: a national-scale analysis of parental race/ethnicity, disparities in prenatal PM_2.5_ exposures and related impacts on birth outcomes

**DOI:** 10.1186/s12940-022-00856-w

**Published:** 2022-05-06

**Authors:** Devon C. Payne-Sturges, Robin Puett, Deborah A. Cory-Slechta

**Affiliations:** 1 School of Public Health, Maryland Institute for Applied Environmental Health, University of Maryland, 255 Valley Drive, College Park, MD 20742 USA; 2 School of Medicine, University of Rochester, Box EHSC, Rochester, NY 14642 USA

**Keywords:** Air pollution, Birth outcomes, Health disparities, Environmental justice, Paternal characteristics, Asian American and Pacific Islanders (AAPI)

## Abstract

**Background:**

Most U.S. studies that report racial/ethnic disparities in increased risk of low birth weight associated with air pollution exposures have been conducted in California or northeastern states and/or urban areas, limiting generalizability of study results. Few of these studies have examined maternal racial/ethnic groups other than Non-Hispanic Black, non-Hispanic White and Hispanic, nor have they included paternal race. We aimed to examine the independent effects of PM_2.5_ on birth weight among a nationally representative sample of U.S. singleton infants and how both maternal and paternal race/ethnicity modify relationships between prenatal PM_2.5_ exposures and birth outcomes.

**Methods:**

We used data from the Early Childhood Longitudinal Study, Birth Cohort (ECLS–B), a longitudinal nationally representative cohort of 10,700 U.S. children born in 2001, which we linked to U.S.EPA’s Community Multi-scale Air Quality (CMAQ)-derived predicted daily PM2.5 concentrations at the centroid of each Census Bureau Zip Code Tabulation Area (ZCTA) for maternal residences. We examined relationships between term birthweight (TBW)_,_ term low birthweight rate (TLBW) and gestational PM_2.5_ pollutant using multivariate regression models. Effect modification of air pollution exposures on birth outcomes by maternal and paternal race was evaluated using stratified models. All analyses were conducted with sample weights to provide national-scale estimates.

**Results:**

The majority of mothers were White (61%). Fourteen percent of mothers identified as Black, 21% as Hispanic, 3% Asian American and Pacific Islander (AAPI) and 1% American Indian and Alaskan Native (AIAN). Fathers were also racially/ethnically diverse with 55% identified as White Non-Hispanic, 10% as Black Non-Hispanic, 19% as Hispanic, 3% as AAPI and 1% as AIAN. Results from the chi-square and ANOVA tests of significance for racial/ethnic differences indicate disparities in prenatal exposures and birth outcomes by both maternal and paternal race/ethnicity. Prenatal PM_2.5_ was associated with reduced birthweights during second and third trimester and over the entire gestational period in adjusted regression models, although results did not reach statistical significance. In models stratified by maternal race and paternal race, one unit increase in PM_2.5_ was statistically significantly associated with lower birthweights among AAPI mothers, -5.6 g (95% CI:-10.3, -1.0 g) and AAPI fathers, -7.6 g (95% CI: -13.1, -2.1 g) during 3^rd^ trimester and among births where father’s race was not reported, -14.2 g (95% CI: -24.0, -4.4 g).

**Conclusions:**

These data suggest that paternal characteristics should be used, in addition to maternal characteristics, to describe the risks of adverse birth outcomes. Additionally, our study suggests that serious consideration should be given to investigating environmental and social mechanisms, such as air pollution exposures, as potential contributors to disparities in birth outcomes among AAPI populations.

**Supplementary Information:**

The online version contains supplementary material available at 10.1186/s12940-022-00856-w.

## Background

Prenatal exposure to PM_2.5_ air pollution has been linked with adverse pregnancy outcomes such as low birth weight (LBW) [1–10]. These outcomes are associated with infant mortality, increased risk for neurodevelopmental delays, hearing impairment, intellectual and developmental disabilities and other health complications which can have lifelong impacts [11–14]. Both rates of LBW [15] and air pollution exposures [16–22] are known to vary by race/ethnicity and socio-economic status. Prior research examining the differential impacts of PM_2.5_ on birth weight by maternal race and socio-economic status have assessed effect modification and/or included interaction terms in regression models (see Additional File 1). Authors of these studies theorize that racialized differences indicate differing susceptibility to ambient air pollution due to increased personal exposure and/or differential biological sensitivity to air pollution stemming from differences in underlying health status, access to health care, or psychosocial stress. The most frequently noted high risk group are Black mothers, however, the direction of association is not always as expected or reaches statistical significance. Bell et al. found the effect of PM_2.5_ on reduced birth weight was stronger among infants of Black mothers (− 22.6 g per IQR, 95% CI: − 29.3, − 15.9) compared to those of white mothers (− 14.7 g, 95% CI: − 17.3, − 12.0) [23]. Morello-Frosch et al. also found that PM_2.5_ effect estimates for decreases in average birth weight were strongest for African Americans [24]. However, Ebisu and Bell found relative risk of LBW associated with IQR increase in PM_2.5_ elemental carbon was 7.3% (95%CI: 4.9, 9.6%) *lower* among African American mothers compared with white mothers [25]. Two other studies also noted counterintuitive results where higher birth weight was associated with increased gestational exposure to PM_2.5_ among racial/ethnic minority mothers [26, 27]. Most of these studies have been conducted in California or northeastern states and/or urban areas, limiting generalizability of study results. Not all studies adjusted for tobacco smoke exposure. Another important gap in the existing literature is that few studies have examined maternal racial/ethnic groups other than Non-Hispanic Black, non-Hispanic White and Hispanic, nor have they included paternal race. To our knowledge only one study [27] examined differential effects of prenatal PM_2.5_ exposure and birth weight by maternal race on a national scale.

Emerging evidence suggests that paternal characteristics affect birth outcomes [28–31]. African American fathers have been found to be associated with pre-term birth and risk of LBW irrespective of maternal race/ethnicity when compared with White fathers [29, 30, 32, 33]. A few studies examined birth outcomes in racially and ethnically discordant couples and found that they have higher rates of pre-term births and LBW compared with racially concordant Non-Hispanic White couples [34–36]. Paternal education has been found to predict birth outcomes over and above maternal socioeconomic indicators, and in some cases, more powerfully than maternal factors [28, 37, 38]. Several pathways have been postulated to explain how paternal factors contribute to adverse birth outcomes and racial disparities, including social and material support, health literacy and psychosocial stress [28, 39]. Additionally, scholars posit that chronic stress associated with everyday interpersonal, institutional and structural racism experienced by both mothers and fathers of color and mixed-race couples has an effect on birth outcomes [29, 39, 40]. However, the potential influence of paternal characteristic on birth outcomes associated with air pollution exposure has not been well studied at a national scale. Additionally, given the increasing interest in cumulative health impacts of multiple stressors on minority populations, a more thorough examination of the role of paternal characteristics in studies of air pollution and birth outcomes is needed.

In our study, we aimed to examine the independent effects of PM_2.5_ on term birth weight among a nationally representative sample of infants. We examined how both maternal and paternal race/ethnicity modify relationships between prenatal PM_2.5_ exposures and birth outcomes. For our study we theorized that race is a proxy measure for exposure to systemic racism that provides the basis of allocating power and privilege to Whites, and disadvantages to racial/ethnic minorities [41]. These interlocking systems of power and policy decisions create harmful living conditions and environmental inequalities for both mothers and fathers through discriminatory practices of residential segregation, in housing, education, employment, criminal justice and health care, and in disproportionate siting of polluting sources in communities of color [17, 42–46]. Social inequalities produced by systemic racism become embodied in the biology of racialized groups and individuals, which we observe as the social patterning of population health outcomes [47, 48], including birth outcomes. We hypothesized that prenatal exposures to PM_2.5_ and relationships with birth outcomes would vary by race/ethnicity of both parents, indicating a cumulative effect of exposure to both air pollution and stress of living in a racialized society.

## Methods

### Study population

Data were from the Early Childhood Longitudinal Study, Birth Cohort (ECLS–B), a longitudinal nationally representative data set that was collected by the National Center for Education Statistics (NCES), U.S. Department of Education to study children’s health, early learning, development, and education experiences [49]. The ECLS-B used a clustered, list frame design to select a nationally representative probability sample of the approximately four million children born in 2001. Registered births were sampled within primary sampling units (counties or groups of contiguous counties) from the National Center for Health Statistics vital statistics system. The survey oversampled very low birth weight (VLBW) babies, multiple births, and Asian and American Indian births [49]. More than 14,000 births were sampled and contacted; from these sampled births, the final study cohort (consisting of completed baseline interviews) of 10,700 was formed when the children were aged approximately nine months [49]. This represents an initial response rate of 76%. The features of the ECLS-B, including multiple measures of gestational, birth, and socio-demographic characteristics make it well-suited to the research questions of the current study. For our study we used data collected at baseline (from when the children were about nine months) which included data from the infant’s birth certificate, computer-assisted personal interviews, and parental self-administered questionnaires.

We obtained restricted data for this study by permission and with approval from the Institute for Education Sciences Data Security Office of the US Department of Education, National Center for Education Statistics (NCES). Sample sizes have been rounded to the nearest 50 in accordance with NCES requirements for ensuring participant confidentiality. This study was reviewed and approved by the NCES and the Institutional Review Board of the University of Maryland.

### Birth data

We restricted our study to singleton, term births (births with gestational period ≥ 37 weeks) because we were interested in fetal growth restriction independent of gestational duration. We excluded births with maternal age > 49 years, unreasonable gestational ages (> 44 weeks) or unreasonable combinations of gestational age and birth weights [50]. Our final analytical sample size was 6,200 births, weighted to be representative of approximately 3.3 million U.S. singleton term births in 2001.

Data collected from birth records included year and month of birth, last menstrual period (LMP); adequacy of prenatal care (the Kessner index adequate, intermediate, and inadequate[51]); mother’s characteristics (age, race/ethnicity, marital status, education, tobacco use during pregnancy); father’s characteristics (age and race/ethnicity); gestational age in weeks; parity; sex of child; and birth weight. For analytical purposes, we used non-Hispanic Black, non-Hispanic White, Hispanic, non-Hispanic Asian American and Pacific Islander (AAPI), non-Hispanic American Indian and Alaskan Native (AIAN) as racial/ethnic categories for mothers and fathers. AAPI included Chinese, Japanese, Hawaiian, Filipino, Asian Indian, Korean, Samoan, Vietnamese, Guamanian and other Asian or Pacific Islanders. Those with paternal race/ethnicity listed as “not stated” were included as an additional racial/ethnic category. Hereafter, Black, White, AAPI and AIAN will be used to denote non-Hispanic ethnicities for these groups. From the 9-month baseline ECLS-B assessment, we extracted residential zip code, and household poverty status (below 185% poverty as determined by ECLS-B using data on household income and household size obtained during the parent interview and the U.S. Bureau of the Census’ weighted poverty thresholds for 2001 for households with children), US Region (Northeast, Midwest, South, West), urbanicity (urban or rural) descriptors for mother’s residence and type of father (resident or non-resident).

Using the estimated date of conception (14 days from last monthly period (LMP)), we calculated trimester divisions of 1–13 weeks, 14–26 weeks, and 27 weeks to birth; similar definitions have been applied elsewhere [23, 26, 51, 52]. We used the estimated date of conception and these trimesters to estimate air pollution exposure for each pregnancy for the total pregnancy and each trimester.

### Exposure assessment

Predicted daily 24-h average PM_2.5_ air concentrations (μg/m^3^) at the geographic centroid of each 2010 U.S. Census zip code tabulation area (ZCTA) for the period January 1, 2001 through December 31, 2001 from the U.S. EPA were used to estimate average daily residential prenatal exposures to PM_2.5_. Thus, births whose gestation extended before Jan 1, 2001 were excluded. The ZCTA residence for study population was based on the zip code of residence at the time of 9-month ECLS-B assessment timepoint. This implies that it was assumed that parents did not move from their residence since the child’s birth. These daily modeled PM_2.5_ concentrations were based on U.S. EPA’s downscaling model which uses a Bayesian space–time modeling approach to combine or “fuse” 12-km gridded outputs from U.S.EPA’s Community Multi-scale Air Quality (CMAQ) model with particulate air monitoring data from US national, state and local air monitoring stations [53–55]. The CMAQ is a validated and widely used model that combines atmospheric transport models with emission models and meteorological models to aid air quality management [56–58]. The “downscaler” tool combines the best attributes of historical air monitoring stations, CMAQ and other models to improve predictive maps of pollution at local scales and has been used to evaluate health impacts associated with PM_2.5_ for CDC’s Public Health Tracking program [59–61].

### Statistical analysis

We determined our covariates and modeling analyses based on previous reports to allow for comparisons [23, 25, 26, 62, 63]. First, we assessed demographics, clinical factors, and other characteristics by term birth weight (TBW), term low birth weight (TLBW) prevalence and gestational PM_2.5_ exposures using Chi-square tests for categorical and ANOVA tests for continuous variables. Second, bivariate models were used to test the associations between our outcomes and PM_2.5_ exposures and covariates. Then we assessed the association between non-pollutant variables and TBW in linear models and TLBW in logistic models. TBW was included as a continuous variable and TLBW was dichotomized as < 2,500 g versus non-low weight ≥ 2,500 g births. TLBW is defined as low birth weight among infants with gestational age ≥ 37 weeks and denotes occurrence of fetal growth restriction.

Non-pollutant models of TBW and TLBW were adjusted for child sex, parity, gestational age, adequacy of prenatal care, maternal age (< 20, 20–24, 25–34, 35–39, > 39 years) accounting for nonlinear relationship between mother’s age and birth weight [64], maternal education (< 12 years, 12 years, 13–15 years, > 15 years and unknown), maternal marital status, maternal and paternal race, father’s age, household poverty status as an indicator of material deprivation, tobacco use during pregnancy (yes/no), and characteristics of residential location (US Region and urbanicity). This allowed us to explore whether expected associations were observed (e.g., maternal smoking associated with lower birth weight). Covariates exhibiting a statistically significant relationship with birth weight were incorporated into models investigating air pollution exposure as in previous studies [23].

We examined relationships between continuous TBW and gestational PM_2.5_ pollutant exposure by trimester, using multivariate regression models assigning exposure averages for each trimester and whole pregnancy. We also assessed the relationships between trimester and whole pregnancy exposures to PM_2.5_ and TLBW. The models were adjusted for statistically significant covariates from non-pollutant models. Sensitivity analyses examined the effects of including parental race in main effects models. Effect modification of air pollution exposures on birth outcomes by maternal and paternal race was evaluated using stratified models. All analyses were conducted with STATA version 14 (StataCorp, College Station, TX) using the “svy” commands and weighted to appropriately account for the oversampling of some population groups and the stratified cluster design of the ECLS-B. Taylor series linearization was used to estimate the standard errors for parameter estimates. All results are based on weighted counts.

## Results

Table 1 shows the (weighted) descriptive statistics for our nationally representative study population of approximately 3.3 million singleton term births born in 2001. Results are consistent with national vital statistics and other reports [15, 61, 65, 66]. Average TBW was 3,423.6 g. Two percent of these term births were LBW infants (< 2,500 g). Fifty-one percent of births were male sex and 41% were first births. Average gestational length was 39.3 weeks. The majority of mothers were married (69%), White (61%) and on average were 27 years old. Overall, 11% of mothers reported smoking during pregnancy. Fourteen percent of mothers identified as Black, 21% as Hispanic, 3% AAPI and 1% AIAN. Fathers were also racially/ethnically diverse with 55% identified as White Non-Hispanic, 10% as Black, 19% as Hispanic, 3% as AAPI and 1% as AIAN. However, for 12% of births, father’s race was “not stated” and among these births, 93.5% were to unmarried mothers (see Additional File 1). Higher rates of household poverty (82%) and less than high school educational attainment of mothers (40%) was observed among infants with father’s race “not stated” on birth certificates compared to the overall sample (47% and 20% respectively). Proportion of births where mothers reported fathers of a different race or ethnic background from their own and/or “not stated” varied: 15%, 40%, 19%, 20% and 70% among White, Black, Hispanic, AAPI and AIAN mothers, respectively (see Additional File 1). In other words, 20% of births were to interracial couples. At the 9-month assessment time point, 81% of infants were living with both biological parents, 18% did not have a father residing and the remaining 1% were residing in households with either adoptive father, stepfather, foster father or mother’s partner. Type of father (resident and non-resident) varied by mother’s race/ethnicity and educational attainment, father’s race/ethnicity, and household poverty status (see Additional File 1).

**Table 1:** Characteristics of U.S. singleton term births born in 2001, Early Childhood Longitudinal Study-Birth Cohort (ECLS-B) study sample

	**% of Sample Births**	**Birth Weight (grams)**	
		mean	sd
**TOTAL**	100%	3423.6	477.7
**Child Sex (%)**			
** Male**	51%	3485.9	478.7
** Female**	49%	3358.2	467.9
**Parity (%)**			
** First child**	41%	3378.6	473.8
** Not first child**	59%	3454.3	478.0
**Gestation (weeks)**	39.3		1.5
**Adequacy of prenatal care (Kessner Index) (%)**			
** Adequate**	78%	3443.7	468.6
** Intermediate**	17%	3355.4	498.0
** Inadequate**	5%	3335.9	463.8
**Mother's education (%)**			
** < 12 years**	20%	3361.5	471.5
** 12 years**	31%	3390.0	482.6
** 13—15 years**	22%	3460.8	467.0
** > 15 years**	26%	3485.9	470.8
** unknown**	1%	3328.4	539.0
**Mother's race/ethnicity**			
** White, non-Hispanic**	61%	3476.2	406.1
** Black, non-Hispanic**	14%	3348.1	500.3
** Hispanic, any race**	21%	3409.6	414.4
** AAPI**^**a**^	3%	3272.0	997.0
** American Indian/Alaskan Native**	1%	3446.3	1490.8
**Fathers 's race/ethnicity**			
** White, non-Hispanic**	55%	3479.9	409.0
** Black, non-Hispanic**	10%	3315.4	478.2
** Hispanic, any race**	19%	3408.3	404.7
** AAPI**^**a**^	3%	3270.1	1048.3
** American Indian/Alaskan Native**	1%	3440.8	1363.7
** Not stated**	12%	3318.5	515.5
**Mother's marital status**			
** Married**	69%	3459.8	464.4
** Not Married**	31%	3344.1	497.3
**Mother's age**			
**Mean (years)**	27.3		6.1
** < 20 yrs (%)**	11%	3318.6	507.5
** 20—24**	26%	3355.0	457.0
** 25—29**	27%	3452.5	454.9
** 30—34**	23%	3481.3	485.2
** 35—39**	11%	3471.3	490.1
** > 39**	2%	3515.1	516.7
**Father's age**			
**Mean (years)**	38.3		22.9
** < 45 yrs (%)**	86%	3439.0	471.0
** ≥ 45**	2%	3401.1	498.2
** Not stated**	12%	3313.1	508.54
**Urbanicity (%)**			
** Rural**	14%	3405.2	476.2
** Urban**	86%	3426.6	477.9
**Region (%)**			
** Northeast**	17%	3432.2	448.5
** Midwest**	23%	3461.2	483.5
** South**	37%	3397.5	466.2
** West**	23%	3421.8	506.9
**Smoked during pregnancy (%)**			
** Yes**	11%	3257.4	452.5
** No**	75%	3449.0	468.2
** Unknown**	14%	3412.6	520.2
**Poverty**			
** < 185% Poverty**	47%	3358.0	482.0
** = > 185%**	53%	3481.1	466.5

Estimates are weighted to take the complex sampling design into account

^a^ Asian American and Pacific Islander (AAPI), includes Chinese, Japanese, Hawaiian, Filipino, Asian Indian, Korean, Samoan, Vietnamese, Guamanian and other Asian or Pacific Islander. Weighted total: *n* = 3,252,800 births; unweighted total: *n* = 6,200 births. Sample size rounded to nearest 50 in accordance with ECLS-B data confidentiality requirement

Mean TBWs differed by all demographic variables as expected, except urbanicity (see Table 1). For example, male infants tended to be heavier than female. Infants born to either mother or father of a racial or ethnic minority group or father’s “race not stated” had lower birth weights compared to White mothers and fathers. Birth weights were lower among mothers living in poverty compared to those who were not. Mothers who reported smoking during pregnancy had lower birth weights compared to non-smoking mothers. Prevalence of TLBW also differed by all demographic characteristics except prenatal care, urbanity and region of the U.S., compared to normal weight births in the sample (see Additional File 1). Rates of TLBW births were disproportionate among African American mothers and fathers, births where father’s race was not stated and households below 185% poverty relative to their proportion in total sample of term births.

As shown in Table 2, average prenatal PM_2.5_ exposures was highest at 15.6 ug/m^3^ during first trimester and lowest during third trimester at 14.4 µg/m^3^. Average PM_2.5_ exposure over the entire pregnancy was 14.9 µg/m^3^ with interquartile range of 4.6 µg/m^3^. Gestational exposures (trimester periods and entire pregnancy) were highly correlated (see Additional File 1). Because of the correlation between trimester exposures, we did not examine models using all trimesters concurrently in this study. During our study period, the US EPA PM2.5 NAAQS Annual Primary and Secondary Standard was 15 µg/m^3^. Fifty percent of births had gestational exposures at or below this health-based standard (see Additional File 1 for the distribution of gestation exposures). Prenatal PM_2.5_ exposures were statistically significantly different by maternal and paternal racial and ethnicity categories, urbanicity and US Region. Babies born to Black and AAPI moms and dads tended to have higher average prenatal PM_2.5_ exposures, although there was overlap of the confidence intervals with other groups.

**Table 2:** Prenatal PM2.5 exposures by trimester and whole pregnancy among the ECLS-B study population

	**PM2.5 (µg/m**^**3**^**) average daily over** **1st trimester** **(95%CI)**	*p*-value	**PM2.5 (µg/m**^**3**^**) average daily over 2nd trimester (95%CI)**	*p*-value	**PM2.5 (µg/m**^**3**^**) average daily over 3rd trimester (95%CI)**	*p*-value	**PM2.5 (µg/m**^**3**^**) average daily over whole pregnancy (95%CI)**	*p*-value
**Total**	15.6 (15.2—16.0)		14.8 (14.4—15.2)		14.4 (14.0—14.9)		14.9 (14.5—15.3)	
**Child Sex (%)**		0.559		0.894		0.204		0.292
** Male**	15.5 (15.0, 16.1)		14.8 (14.3, 15.3)		14.3 (13.9, 14.8)		14.86 (14.4, 15.3)	
** Female**	15.7 (15.3, 16.1)		14.8 (14.3, 15.2)		14.6 (14.1, 15.0)		15.0 (14.6, 15.4)	
**Parity (%)**		0.499		**0.047**		0.870		0.306
** First child**	15.7 (15.2, 16.2)		15.0 (14.6, 15.4)		14.5 (14.1, 14.9)		15.0 (14.6, 15.4)	
** Not first child**	15.5 (15.1, 16.0)		14.6 (14.2, 15.1)		14.4 (13.9, 14.9)		14.9 (14.4, 15.3)	
**Adequacy of prenatal care (Kessner Index) (%)**		0.928		0.651		0.991		0.975
** Adequate**	15.6 (15.2, 16.0)		14.8 (14.4, 15.3)		14.4 (14.0, 14.9)		14.9 (14.5, 15.3)	
** Intermediate**	15.5 (14.8, 16.2)		14.7 (14.1, 15.3)		14.4 (13.9, 15.0)		15.0 (14.5, 15.4)	
** Inadequate**	15.6 (14.3, 16.9)		14.4 (13.4, 15.5)		14.5 (13.4, 15.5)		14.9 (14.0, 15.8)	
**Maternal education (%)**		0.196		0.586		**0.010**		**0.005**
** < 12 years**	15.8 (15.2—16.3)		15.0 (14.4—15.7)		15.0 (14.4- 15.6)		15.3 (14.9—15.9)	
** 12 years**	15.5 (15.0—16.0)		14.8 (14.3—15.2)		14.4 (13.9—14.9)		14.9 (14.4—15.3)	
** 13—15 years**	15.6 (15.0—16.2)		14.7 (14.1—15.3)		14.4 (13.8—14.9)		14.9 (14.4—15.4)	
** > 15 years**	15.6 (14.9—16.2)		14.7 (14.1—15.3)		14.1 (13.6—14.6)		14.7 (14.3—15.2)	
** unknown**	16.2 (14.0—18.4)		14.9 (13.3—16.6)		14.5 (13.2—15.8)		14.9 (14.0—15.8)	
**Maternal race/ethnicity**		**0.000**		**0.000**		**0.000**		**0.000**
** White, non-Hispanic**	15.3 (14.8, 15.7)		14.6 (14.2, 15.0)		14.1 (13.7, 14.6)		14.7 (14.3, 15.1)	
** Black, non-Hispanic**	15.6 (15.0, 16.1)		15.3 (14.7, 15.9)		15.1 (14.4, 15.7)		15.4 (14.8, 15.9)	
** Hispanic, any race**	16.5 (15.4, 17.6)		15.0 (14.2, 15.8)		14.9 (14.1, 15.7)		15.4 (14.6, 16.2)	
** AAPI**^a^	16.6 (15.9, 17.3)		15.2 (14.7, 15.7)		15.1 (14.6, 15.5)		15.6 (15.2, 16.0)	
** American Indian/Alaskan Native**	13.9 (12.8, 15.0)		12.7 (12.0, 13.3)		12.1 (11.5, 12.7)		12.7 (12.2, 13.3)	
**Paternal race/ethnicity**		**0.030**		**0.024**		**0.000**		**0.000**
** White, non-Hispanic**	15.2 (14.8, 15.7)		14.6 (14.2, 15.1)		14.2 (13.7, 14.6)		14.7 (14.3, 15.1)	
** Black, non-Hispanic**	15.5 (14.9, 16.2)		15.0 (14.4, 15.7)		15.1 (14.4, 15.8)		15.4 (14.7, 16.0)	
** Hispanic, any race**	16.6 (15.6, 17.6)		15.0 (14.2, 15.9)		14.9 (14.1, 15.7)		15.4 (14.6, 16.2)	
** AAPI**^a^	16.4 (15.6, 17.1)		15.3 (14.8, 15.9)		15.1 (14.6, 15.5)		1.7 (15.2, 16.1)	
** American Indian/Alaskan Native**	14.3 (13.3,15.3)		13.8 (13.2, 14.5)		12.9 (12.4, 13.3)		13.4 (13.0, 13.8)	
** Not stated**	15.4 (14.9, 16.0)		14.9 (14.3, 15.4)		14.4 (13.9, 14.9)		14.8 (14.4, 15.3)	
**Maternal marital status**		0.642		0.080		0.073		0.111
** Married**	15.6 (15.0, 16.1)		14.7 (14.2, 15.1)		14.3 (13.9, 14.8)		14.8 (14.4, 15.3)	
** Not Married**	15.7 (15.2, 16.2)		15.0 (14.6, 15.5)		14.6 (14.2, 15.1)		15.1 (14.7, 15.6)	
**Mother's age**		0.421		0.984		0.105		0.473
** < 20 yrs (%)**	15.2 (14.4, 15.9)		14.8 (14.2, 15.3)		14 = 5.0 (14.4, 15.5)		15.1 (14.6, 15.6)	
** 20—24**	15.6 (15.1, 16.1)		14.8 (14.3,15.3)		14.2 (13.8, 14.7)		14.8 (14.3, 15.2)	
** 25—29**	15.5 (14.9, 16.1)		14.8 (14.4, 15.3)		14.5 (13.9, 15.0)		14.9 (14.48, 15.41)	
** 30—34**	16.00 (15.4, 16.6)		14.8 (14.3, 15.3)		14.4 (13.9, 14.9)		15.0 (14.5, 15.4	
** 35—39**	15.4 (14.6, 16.3)		14.6 (13.9, 15.4)		14.4 (13.7, 15.0)		14.9 (14.3, 15.6)	
** > 39**	15.9 (14.5, 17.4)		14.6 (13.8, 15.4)		14.6 (13.2, 16.0)		15.2 (13.9, 16.5)	
**Paternal age**		0.227		**0.010**		0.951		0.795
** < 45 yrs (%)**	15.7 (15.2, 16.1)		14.80 (14.4, 15.2)		14.4 (14.0, 14.9)		14.9 (14.5, 15.4)	
** ≥ 45**	14.7 (13.6, 15.8)		13.6 (12.6, 14.5)		14.3 (13.2, 15.4)		14.7 (13.8, 15.6)	
** Not stated**	15.4 (14.8, 15.9)		15.0 (14.4, 15.6)		14.4 (13.9, 15.0)		14.9 (14.4, 15.3)	
**Urbanicity (%)**		**0.0001**		**0.018**		0.126		**0.016**
** Rural**	14.5 (13.9, 15.2)		14.2 (13.6, 14.7)		14.0 (13.4, 14.6)		14.3 (13.8, 14.9)	
** Urban**	15.8 (15.3, 16.3)		14.9 (14.4, 15.3)		14.5 (14.0, 15.0)		15.0 (14.6, 15.5)	
**Region (%)**		**0.000**		**0.000**		**0.011**		**0.000**
** Northeast**	15.6 (15.0, 16.2)		15.0 (14.6, 15.3)		14.4 (14.0, 14.8)		15.0 (14.7, 15.4)	
** Midwest**	17.0 (16.6, 17.6)		16.2 (15.7, 16.7)		15.4 (14.9, 16.0)		16.4 (15.9, 16.8)	
** South**	14.1 (13.3, 14.9)		14.3 (13.3, 15.2)		14.0 (13.0, 14.9)		14.1 (13.2, 14.9)	
** West**	16.6 (15.4, 17.8)		14.1 (13.3, 15.0)		14.3 (13.4, 15.2)		14.9 (13.9, 15.8)	
**Smoked during pregnancy (%)**		0.601		0.813		0.225		0.386
** Yes**	15.0 (14.5, 15.4)		14.6 (14.0, 15.1)		14.2 (13.7, 14.7)		14.7 (12.2, 15.1)	
** No**	15.1 (14.6, 15.6)		14.5 (14.0, 15.0)		14.0 (13.5, 14.5)		14.5 (14.1, 14.9)	
**Poverty**		0.393		0.361		0.139		0.375
** < 185% Poverty**	15.7 (15.2, 16.2)		14.9 (14.5, 15.3)		14.6 (14.1, 15.1)		15.0 (15.0, 15.5)	
** = > 185%**	15.5 (15.0, 16.0)		14.7 (14.2, 15.2)		14.3 (13.8, 14.8)		14.9 (14.4, 15.3)	

All analyses conducted with sample weights

^a^ Asian American and Pacific Islander (AAPI), includes Chinese, Japanese, Hawaiian, Filipino, Asian Indian, Korean, Samoan, Vietnamese, Guamanian and other Asian or Pacific Islander

Tables 3 and 4 show unadjusted and adjusted multivariable analyses. In comparison with White mothers, all other groups on average, had lower birth weights with Black and AAPI moms showing the greatest differences, -228.1 g and -204.2 g respectively. Similar birth weight disparities were observed among newborns by race/ethnicity of fathers, with the largest difference among AAPI fathers. In Model 1 (Table 3), examining the associations between non-pollutant variables and term birth weight without race, we found significant positive associations with sex, gestational length and parity. On average male babies and not first-in-birth-order babies were heavier. Inadequate prenatal care, younger ages of mothers, smoking during pregnancy and poverty were statically significantly associated with lower birth weights. After adding maternal and paternal race separately to these models (Models 2 and 3), these associations were unchanged. We observed that in these separate models, Black, Hispanic and AAPI fathers were associated with lower birth weight babies, with similar effect sizes as Black, Hispanic and AAPI mothers. However, in Model 4 with both maternal and paternal race, only lower birth weights associated AAPI fathers remained statistically significant.

**Table 3:** Difference in birth weight associated with selected non-pollutant variables among the ECLS-B study population, crude and adjusted models

	Crude	**Model 1**	**Model 2**	**Model 3**	**Model 4**
		**Non-pollutants associations (no race)**	**Non-pollutants associations (maternal race only)**	**Non-pollutants associations (paternal race and age only)**	**Non-pollutants associations (maternal and paternal race)**
	Birth Weight (grams)	Birth Weight (grams)	Birth Weight (grams)	Birth Weight (grams)	Birth Weight (grams)
**Parameter**	β Estimate (95% CI)	β Estimate (95% CI)	β Estimate (95% CI)	β Estimate (95% CI)	β Estimate (95% CI)
** Male**	127.7 (103.7, 151.7)	124.0 (101.0, 147.0)	122.9 (100.2, 145.6)	124.0 (101.6, 146.4)	122.8 (100.1, 145.5)
** Female**	reference	reference	reference	reference	reference
**Parity**					
** First child**	reference	reference	reference	reference	reference
** Not first child**	75.7 (46.3, 105.0)	88.1 (54.3, 121.9)	90.3 (56.3, 124.2)	87.8 (54.0, 121.6)	90.1 (56.3, 124.0)
**Adequacy of prenatal care (Kessner Index)**					
** Adequate**	reference	reference	reference	reference	reference
** Intermediate**	-88.4 (-128.9, -47.8)	-58.3 (-96.1, -20.5)	-47.4 (-84.2, -10.6)	-51.7 (-89.7, -13.8)	-47.5 (-84.2, -10.8)
** Inadequate**	-107.8 (-171.8, -43.9)	-63.1 (-127.2, 1.1)	-45.6 (-112., 20.9)	-56.8 (-122.9, 9.3)	-46.1 (-112.1, 19.8)
** Gestational length (weeks)**	60.2 (51.3, 69.0)	65.8 (56.6, 74.9)	64.9 (55.9, 74.0)	65.8 (56.7, 74.8)	64.9 (55.9, 73.9)
**Maternal education**					
** < 12 years**	reference	reference	reference	reference	reference
** 12 years**	28.5 (-11.8, 68.8)	-13.6 (-56.5, 29.3)	-6.0 (-52.2, 40.2)	-13.5 (-57.8, 30.8)	-6.3 (-51.9, 39.4)
** 13—15 years**	99.3 (49.9, 148.8)	-1.3 (-46.6, 4406)	1.2 (-48.3, 50.7)	-1.8 (-49.5, 45.9)	0.4 (-48.8, 49.5)
** > 15 years**	124.4 (81.8, 167.1)	-22.6 (-66.9, 21.72)	-22.1 (-70.7, 26.6)	-24.1 (-71.7, 23.5)	-22.5 (-71.8, 26.7)
** unknown**	-33.1 (-142.8, 76.5)	-98.5 (-208.0, 11.0)	-75.6 (-189.0, 37.8)	-83.7 (-196.0, 28.6)	-81.1 (-194.7, 32.5)
**Maternal race/ethnicity**					
** White**	reference		reference		reference
** Black**	-228.1 (-262.4, -193.8)		-202.9 (-238.8, 167.1)		-222.1 (-277.6, -166.5)
** Hispanic**	-66.6 (-107.4, -25.7)		-53.1 (-100.2, -6.1)		-39.6 (-95.5, 16.3)
** AAPI**^a^	-204.2 (-237.5, -170.9)		-216.1 (-252.2, -180.1)		-146.6 (-211.6, -81.7)
** American Indian/Alaskan Native**	-29.9 (-99.2, 30.4)		13.6 (-56.2, 83.5)		14.2 (-62.5, 90.9)
**Paternal race/ethnicity**					
** White**	reference			reference	reference
** Black**	-164.5 (-202.2, -126.8)			-143.9 (-188.9, -98.9)	30.1 (-34.3, 94.5)
** Hispanic**	-71.6 (-108.0, -35.2)			-45.2 (-91.5, 1.2)	-17.98 (-71.8, 35.9)
** AAPI**^a^	-209.7 (-245.6, -173.9)			-215.30 (-251.41, -179.19)	-93.7 (-159.8, -27.7)
** American Indian/Alaskan Native**	-39.0 (-93.2, 15.1)			1.6 (-55.3, 58.4)	5.3 (-58.2, 68.9)
** Not stated**	-161.4 (-203.7, -119.1)			-27.7 (-127.8, 72.5)	34.5 (-66.6, 135.7)
**Maternal marital status**					
** Married**	reference	reference	reference	reference	reference
** Not Married**	-115.7 (-147.1, -84.3)	-28.1 (-65.1, 8.9)	1.8 (-37.0, 40.5)	-5.5 (-47.4, 36.4)	-1.7 (-43.0, 39.7)
**Maternal age (years)**					
** < 20 yrs**	-162.7 (-219.6, -105.9)	-53.6 (-113.0, 5.8)	-54.9 (-115.8, 6.1)	-57.3 (-117.4, 2.8)	-53.8 (-115.2, 7.6)
** 20—24**	-126.3 (-167.8, -84.8)	-66.4 (-110.9, -21.8)	-70.6 (-114.7, -26.5)	-68.3 (-112.8, -23.7)	-70.0 (-114.1, -25.9)
** 25—29**	-28.8 (-73.8, 16.2)	-15.0 (-56.9, 27.0)	-10.9 (-52.7, 30.9)	-11.2 (-53.3, 31.0)	-10.6 (-52.4, 31.2)
** 30—34**	reference	reference	reference	reference	reference
** 35—39**	-10.0 (-54.7, 34.7)	-2.1 (-43.4, 39.3)	-1.5 (-42.8, 39.8)	-3.0 (-43.8, 37.8)	-1.8 (-43.2, 39.6)
** > 39**	33.8 (-53.7, 121.3)	32.2 (-64.2, 128.6)	33.3 (-64.0, 130.50)	33.1 (-61.9, 128.1)	32.8 (-63.7, 129.3)
**Paternal age (years)**					
** < 45 yrs**	reference	reference	reference	reference	reference
** ≥ 45**	-37.9 (-131.7, 56.0)	-71.8 (-163.9, 20.4)	-48.0 (-139.07, 43.10)	-64.1 (-155.3, 27.1)	-50.7 (-141.6, 40.3)
** Not stated**	-125.9 (-169.2, -82.6)	27.4 (-79.5, 24.6)	0.1 (-51.8, 52.0)	-52.5 (-159.1, 54.1)	-24.7 (-133.6, 84.1)
**Urbanicity**					
** Rural**	reference	reference	reference	reference	reference
** Urban**	21.4 (-21.3, 64.2)	-8.4 (-53.4, 36.7)	22.3 (-23.6, 68.1)	12.8 (-12.5, 58.2)	22.4 (-23.8, 68.6)
**Region**					
** Northeast**	reference	reference	reference	reference	reference
** Midwest**	29.1 (-38.4, 96.6)	27.4 (-25.5, 80.2)	20.0 (-29.5, 69.4)	20.2 (-29.2, 69.7)	19.7 (-29.6, 69.0)
** South**	-34.6 (-97.2, 27.9)	-20.3 (-66.4, 25.7)	-12.7 (-55.8, 30.3)	-17.3 (-61.4, 26.7)	-13.7 (-56.9, 29.6)
** West**	-10.4 (-73.8, 53.0)	12.4 (-41.3, 66.1)	-5.3 (-55.7, 45.1)	4.4 (-46.6, 55.4)	-5.7 (-55.8, 44.5)
**Smoked during pregnancy**					
** Yes**	-191.5 (-233.0, -150.04)	-159.4 (-199.5, -119.3)	-195.4 (-236.8, -154.0)	-175.8 (-215.9, -135.8)	-196.4 (-238.0, -154.7)
** Unknown**		-45.3 (-97.1, 6.6)	-24.4 (-74.7, 25.9)	28.8 (-80.4, 22.8)	-21.0 (-71.1, 29.2)
** No**	reference	reference	reference	reference	reference
**Poverty**					
** < 185% Poverty**	-123.1 (-151.0, -95.3)	-69.0 (-101.6, -36.4)	-46.3 (-78.4, -14.2)	-52.3 (-85.0, -19.7)	-46.4 (-78.5, -14.4)
** = > 185%**	reference	reference	reference	reference	reference

All analyses conducted with sample weights

^a^ Asian American and Pacific Islander (AAPI), includes Chinese, Japanese, Hawaiian, Filipino, Asian Indian, Korean, Samoan, Vietnamese, Guamanian and other Asian or Pacific Islander

**Table 4:** Difference in birth weight associated with prenatal PM2.5 exposures among the ECLS-B study population, crude and adjusted models

	**Crude**	**Model 5**	**Model 6**	**Model 7**	**Model 8**
		**(No race)**	**(Maternal race only)**	**(Paternal race and age only)**	**(Maternal and paternal race)**
**Parameter**	Birth Weight (grams)	Birth Weight (grams)	Birth Weight (grams)	Birth Weight (grams)	Birth Weight (grams)
	β Estimate (95% CI)	β Estimate (95% CI)	β Estimate (95% CI)	β Estimate (95% CI)	β Estimate (95% CI)
**PM2.5**					
** Trimester 1**	0.7 (-3.3—4.6)	0.4 (-3.5, 4.3)	0.8 (-3.2, 4.9)	0.5 (-3.5, 4.5)	0.8 (-3.2, 4.8)
** Trimester 2**	-1.1 (-5.2, 3.0)	-1.0 (-4.8, 2.9)	-0.1 (-3.8, 3.6)	-0.6 (-4.4, 3.1)	-0.04 (-3.8, 3.7)
** Trimester 3**	-2.4 (-5.8, 1.1)	-1.9 (-5.2, 1.5)	-0.7 (-4.0, 2.6)	-1.2 (-4.5, 2.2)	-0.7 (-4.0, 2.6)
** Whole Pregnancy**	-2.1 (-6.1, 2.0)	-2.0 (-6.01, 2.07)	-0.7 (-4.6, 3.1)	-1.2 (-5.2, 2.7)	-0.7 (-4.6, 3.1)

All analyses conducted with sample weights. Models adjusted for covariates that were statistically significant < 0.05 in the non-pollutant models from Table 3: gender, parity, adequacy of prenatal care, gestation, maternal age, smoking and poverty

In our sample, the odds of TLBW were nearly 3.0, 1.54 and 1.84 times higher, respectively, among Black, AAPI and AIAN moms relative to White mothers. Again, similar disparities were observed by paternal race. Increased odds of TLBW were associated with child sex, parity, gestation length, maternal marital status, region, tobacco use during pregnancy and poverty in non-pollutant multivariate models (see Additional File 1). Once adjusting for maternal and paternal race in separate models, these associations were unchanged. Black and AAPI mothers and fathers were independently associated with TLBW. With maternal and paternal race in the same model, only maternal race remained as a significant predictor of TLBW.

Model results of the associations between prenatal PM_2.5_ exposures and term birth weight unadjusted and adjusted for statistically significant covariates from the non-pollutant models in Table 3 (gender, parity, adequacy of prenatal care, gestation, maternal age, smoking and poverty) with and without paternal and maternal race are presented in Table 4. One unit increases in gestational PM_2.5_ exposures, except for first trimester, were associated with decreases in birth weights, but did not reach statistical significance. In adjusted models, PM_2.5_ effect sizes attenuated the association, while Black maternal race and Black and AAPI paternal race relationships remained statistically significant. Similarly, PM_2.5_ was not associated with TLBW, except first trimester exposures which were associated with lower risk; ORs were less than one (see Additional File 1).

We examined the potential effects of race/ethnicity of mother and father separately on the association between gestational PM_2.5_ exposures and TBW (continuous) as well as TLBW using stratified models (Table 5 and Additional File 1). The birth weight impacts of air pollution varied by maternal race and paternal race. In models (See Table 5) stratified by maternal race, a negative and statistically significant association of lower birth weight with higher third trimester PM_2.5_ exposures was seen only among AAPI mothers, -5.6 g (95%CI: -10.3, -1.0). A positive association was noted for AIAN mothers for first trimester exposures. In models stratified by father’s race, we observed strong negative impacts of PM_2.5_ exposures on birth weight among AAPI fathers and especially among fathers whose race “was not stated” on birth certificates. Incorporating father’s race into models stratified by maternal race did not alter the effect sizes of PM_2.5_ on birth weight, and the statistically significant negative association for AAPI mothers and the positive association for AIAN mothers were unchanged. However, we noted that father’s race was independently associated with lower birth weight in models stratified by maternal race. For example, Black and AAPI fathers were independently associated with heavier (β = 113 g, p-value 0.048) and lower (β = -99 g, p-value = 0.061) birth weights, respectively, among White Mothers. AIAN Fathers were independently associated with heavier babies (β = 427 g, p-value = 0.011) among AA Black mothers.

**Table 5:** Stratified models by maternal and paternal race/ethnicity on difference in birth weight associated with prenatal PM2.5 exposures among the ECLS-B study population

Parameter	Birth Weight (grams)	Birth Weight (grams)	Birth Weight (grams)	Birth Weight (grams)	Birth Weight (grams)	Birth Weight (grams)
	β Estimate (95% CI)	β Estimate (95% CI)	β Estimate (95% CI)	β Estimate (95% CI)	β Estimate (95% CI)	β Estimate (95% CI)
**Maternal Race/Ethnicity**						
	White	Black	Hispanic	AAPI^a^	AI/AN	
**PM2.5**						
Trimester 1	-0.5 (-6.0, 5.0)	2.5 (-6.9, 12.0)	1.5 (-6.7, 9.7)	3.5 (-1.9, 8.9)	16.5 (2.00, 31.0)	
Trimester 2	-1.2 (-6.9, 4.6)	-1.8 (-10.6, 7.1)	1.5 (-4.33, 7.29)	-0.6 (-6.5, 5.3)	3.1 (-12.1,18.2)	
Trimester 3	-2.7 (-7.4, 1.9)	-0.9 (-8.3, 6.4)	2.5 (-2.6, 7.7)	-5.6 (-10.3, -1.)	-1.6 (-17.9,14.7)	
Whole Pregnancy	-2.9 (-8.6, 2.7)	-2.7 (-11.5, 6.1)	2.9 (-2.4, 8.2)	-4.0 (-9.1, 1.2)	0.2 (-18.9, 19.3)	
**Paternal Race/Ethnicity**						
	White	Black	Hispanic	AAPI^a^	AI/AN	Not Stated
**PM2.5**						
Trimester 1	-1.4 (-7.3,4.5)	5.3 (-6.8, 17. 5)	1.5 (-6.8, 9.8)	1.5 (-5.0, 7.9)	4.2 (-4.0, 12.5)	1.0 (-13.7, 15.7)
Trimester 2	-0.5 (-6.7, 5.7)	-0.2 (-11.7, 11.3)	1.2 (-5.3, 7.7)	2.6 (-3.6, 8.9)	8.6 (-2.8, 20.0)	-13.9 (-24.3, -3.5)
Trimester 3	-2.9 (-7.9, 2.1)	5.0 (-3.9, 14.0)	3.0 (-2.1, 8.1)	-7.6 (-13.1, -2.1)	5.6 (-6.5, 17.7)	-11.2 (-19.6, -2.9)
Whole Pregnancy	-2.6 (-8.6, 3.5)	4.0 (-6.5, 14.6)	2.6 (-2.8, 8.0)	-5.8 (-12.1, 0.4)	6.9 (-8.2, 22.0)	-14.2 (-24.0, -4.4)
**Maternal Race/Ethnicity**^b^						
	White	Black	Hispanic	AAPI^a^	AI/AN	
**PM2.5**						
Trimester 1	-0.6 (-6.0, 4.8)	2.0 (-7.2, 11.4)	1.8 (-6.6, 10.1)	3.6 (-1.6, 8.8)	16.8 (3.5, 30.1)	
Trimester 2	-1.1 (-6.8, 4.7)	-1.9 (-10.7, 6.9)	1.7 (-4.2, 7.6)	-0.2 (-5.8, 5.5)	3.2 (-12.4, 18.8)	
Trimester 3	-2.8 (-7.5, 1.8)	-1.3 (-8.7, 6.1)	2.5 (-2.6, 7.7)	-5.2 (-9.8, -0.7)	-0.3 (-17.1, 16.6)	
Whole Pregnancy	-3.0 (-8.7, 2.6)	-3.2 (-12.1, 5.7)	3.0 (-2.3, 8.2)	-3.3 (-8.4,1.8)	1.7 (-17.8, 21.2)	

All analyses conducted with sample weights. Models adjusted for covariates that were statistically significant < 0.05 in the non-pollutant models from Table 3: gender, parity, adequacy of prenatal care, gestation, maternal age, smoking and poverty

^a^Asian American and Pacific Islander (AAPI), includes Chinese, Japanese, Hawaiian, Filipino, Asian Indian, Korean, Samoan, Vietnamese, Guamanian and other Asian or Pacific Islander

^b^Also adjusted for paternal race/ethnicity

Overall, we observed no association of gestational PM_2.5_ exposures with TLBW in models stratified by maternal race, except for Hispanic moms during 1st trimester (see Additional File 1). Hispanic mothers had lower risk of TLBW with increasing PM_2.5_. However, in models stratified by paternal race, results were mixed. Among White and Hispanic fathers, PM_2.5_ exposure had a protective effect during second trimester as it did for first trimester exposures among API fathers. Whereas for fathers with "race/ethnicity not stated", risk for TLBW was elevated, with a 10% increase for every unit increase in PM_2.5_ (AOR 1.10, p-value 0.037). In models stratified by maternal race, but also adjusted for paternal race, the only statistically significant association with PM_2.5_ were lower odds of TLBW for first trimester exposures among Hispanic moms. Yet paternal race remained statistically significantly associated with increased TLBW risk in these models.

## Discussion

Our study examined the effects of prenatal PM_2.5_ exposure on birthweight in a nationally representative study of singleton term births in 2001. We explored impacts of pollutant exposure by trimester and effect modification by parental race/ethnicity on these associations. We observed disparities in prenatal exposures by both maternal and paternal race. Both maternal and paternal race/ethnicity were independently associated with birth outcomes in non-pollutant models. We found prenatal PM_2.5_ was associated with reduced birthweights during second and third trimester and over the entire gestational period in adjusted models, although results did not reach statistical significance. As noted by other researchers, the specific gestational window of PM2.5 effects on fetal growth has not been consistent across studies [52, 67, 68]. The reasons for the inconsistences may be due to the different methods researchers have used to consider (or not consider) correlated exposures among trimesters or pollutants [68]. In models stratified by maternal race and paternal race, PM_2.5_ was statistically significantly associated with lower birthweights among AAPI mothers and fathers and among births where father’s race was not reported. We also noted elevated risk of TLBW due to 3^rd^ trimester and whole pregnancy exposures to particulate matter among births where father’s race was not reported. We find these findings about father's race intriguing and worth exploring further in future research on environmental exposures and birth outcomes.

In our stratified analyses, we found comparable results of PM_2.5_ effects on birth weight for White and Black mothers but stronger results for AAPI mothers and fathers. These findings are novel compared to previous studies. Black and Hispanic mothers have been previously reported to have greater reductions in birth weight [23, 69–71] or risk of TLBW [19, 65, 72] due to PM_2.5_ exposures. However, this relationship may depend on spatial scale of exposure assessment and geographic location of study. For example, comparing PM_2.5_ exposure estimates based on county-level average, 5- mile and 1 mile radii from nearest monitor, Basu et al. 2004 found a stronger negative association with birthweight than the neighborhood-monitored air pollutant data for both the non-Hispanic white [5 mile: β for CBW (g) per 1 mg/m^3^ increase in PM_2.5_ = -1.52 (95% confidence interval: -3.52, 0.48), County: β = -4.04 ( -6.71, -1.37)] and Hispanic sample populations in California [5 mile: β = -2.49 ( -4.53, -0.45), County: β = -4.35 ( -7.47, -1.23)] [62]. These estimates found for the Hispanic population suggest a slightly stronger association between PM_2.5_ and birth weight compared to those found for the non-Hispanic white population. This difference, however, was not statistically significant. Using data from monitors within 1 mile of the mother’s residence, stronger associations were found for the non-Hispanic White population compared to the Hispanic population. In a later study using zip code derived PM_2.5_ exposure estimates, results showed Asians, Blacks, and Hispanics, compared to Whites, exhibited *smaller* birth weight reductions for most PM_2.5_ constituents [63]. However, Black and Asian mothers had greater reductions in birth weight with increases in exposure to total PM_2.5_ mass compared to White and Hispanic mothers [ CBW (g) per 1 µg/m^3^ PM_2.5_ (95% CI) Hispanic: -0.74344 (-1.14668, -0.34019); White: -0.83862 (-1.41021, -0.26702); Asian: -1.14198 (-2.05802, -0.22594); Black: -1.05944 (-2.18066, 0.061783)][63]. Similarly, Morello-Frosch and colleagues found changes in birth weight (g) per unit of 10 µg/m3 PM_2.5_ assessed at zip code level were strongest for Black mothers and lowest for Hispanic mothers in California ( -24 g for Black mothers; -15 g for white mothers; -10 g for Hispanics; -14 g for Asian/Pacific Islanders)[24]. A more recent analysis of California births noted effect modification by maternal race/ethnicity and education, with the *lowest* risk of TLBW and lowest birth weight reductions associated with PM_2.5_ total mass exposures found among and Asian mothers and mothers with college level education [65]. Other studies have found counterintuitive results where *higher* birth weight was associated with increase in gestational exposure to PM_2.5_. For example, a study of term births in Texas found heavier babies were associated with prenatal exposure to PM_2.5_ for Hispanic and Black mothers, but not White mothers [26]. Relative risk of LBW associated with IQR increase in PM_2.5_ elemental carbon was 7.3% (95%CI: 4.9, 9.6%) *lower* among African American mothers compared with white mothers [25]. The one national-scale study that assessed effect modification by race found significant association between county level PM_2.5_ and race/ethnicity with all associations *positive* [27], meaning that higher pollution was associated with higher birth weights.

Our findings of higher birthweight reductions among AAPI mothers and fathers add to the literature on perinatal outcome disparities among Asian parents [29, 73, 74]. As noted above, Black and Hispanic mothers have been previously reported to have greater PM_2.5_- related reductions in birth weight or risk of TLBW [23, 69–71]. Whereas PM_2.5_ effects reported for AAPIs has been mixed [24, 65, 75]. The strong effect of air pollution for AAPIs observed in this analysis may be a reflection of the unique characteristics of our sample. For our study we used birth certificate data from the nationally representative ECLS-B. The number of mothers and fathers identifying as AAPIs in our sample was small (1,100 API mothers and 1,000 fathers), although weighted to provide national estimates. If we assume what we observed was real, we might speculate the following about the findings regarding AAPI mothers and fathers. Whites and Blacks are more geographically dispersed, whereas AAPIs tend to cluster in urban areas where we know air pollution is high [76]. AAPIs are less represented in rural communities [77]. In our sample, AAPI mothers and fathers were more likely to be resident in Western regions of the U.S. (45–46%), consistent with national surveys [78]. Additionally, 98% of the AAPI study sample lived in urban areas. The PM_2.5_ exposures were higher among AAPIs compared to other population groups in our sample (Table 2) possibly indicating a nonlinear effect. Other possible explanations may be the difference in chemical composition and corresponding toxicity of PM_2.5_ as result of geographic location [19] and synergistic effects of unmeasured co-exposures to other environmental pollutants and social or psychosocial stress [43, 79].

Studies investigating racial disparities in birthweight have found increased risk of LBW and small for gestational age (SGA) among Asian mothers and couples compared to White mothers and couples [29, 32, 74, 80]. Some researchers attribute the smaller size of babies born to Asian mothers to genetics, since risk for SGA remains elevated after adjusting for “key social variables” such as education and marital status [32]. Yet when disaggregation is possible, data show that birth outcomes significantly differ among AAPI groups [73, 81, 82]. Multiple scientific professional societies have concluded that racial categories are social constructs, products of historical and contemporary social, economic, educational, and political circumstances, and not a fixed biological characteristic [47, 83–85]. Thus, results from our study, together with previous studies, suggest that serious consideration should be given to investigating environmental and social mechanisms, such as air pollution exposures, social class, discrimination, stress, health care access, etc. as contributors to disparities in birth outcomes among AAPI populations [80, 86–89]. Environmental health disparities experienced by Asian and Pacific Islander Americans have been de-emphasized in much prior environmental justice research [90]. Our work adds support for calls for more attention to these injustices by the public health research community.

Existing evidence supports the finding of paternal race/ethnicity to be associated with pregnancy outcomes, independent of maternal race and ethnicity [29, 30, 33]. For example, a study using U.S. Natality data from 1989 to 2013 found that paternal Black race was associated with pre-term birth irrespective of maternal race compared to births of White fathers [32]. A few studies examining birth outcomes in racially and ethnically discordant couples found that they have higher rates of pre-term birth and low birth weight compared with racially concordant White couples [34, 91–93]. Therefore, both maternal and paternal race/ethnicity characteristics should be considered when examining the incidence of adverse pregnancy outcomes. However, the contribution of paternal race/ethnicity, including unreported race, has not been widely examined in studies of (differential) effects of air pollution impacts on birth outcomes. One study in the northeastern region of the U.S. didn’t include paternal race because mother’s and father’s race were found to be highly correlated and that 86.3% of mother–father pairs had the same race classification[23]. However, that may not be case in other regions or at the national scale. Twenty percent of term births in our study were to parents who were not of the same race/ethnicity. In models stratified by paternal education, Enders and colleagues found risk of TBLW associated with course fraction (PM_2.5 – 10_) was enhanced for fathers without a college degree [75]. However, subgroup analysis by paternal race was not examined in that study. In our study, we examined disparities in exposure, outcome and effect modification by paternal race, consistent with guidance on racial health disparities research by Ward et al. [94]. Babies born to Black and AAPI moms and dads tended to have higher average prenatal PM_2.5_ exposures. Relative to White parents, racial and ethnic minority identity was associated with lower birth weights and increased odds of TLBW. We found that paternal race remained independently associated with birth weight, above and beyond maternal race (Models 4 and 8) suggesting paternal factors may be important to consider in investigations of birth outcomes. In stratified models by paternal race, Black, White, AIAN race and Hispanic ethnicity had favorable effects on PM_2.5_-birthweight relationship. Infants whose father’s race was “not stated” or API had significant BW reductions. The finding about father’s race “not stated” is in line with previous studies showing infants whose paternal race and ethnicity were unreported on birth certificates have higher rates of low birthweight, preterm birth and small for gestational age, regardless of maternal race/ethnicity [31, 95–97]. Air pollution exposures may compound an existing risk for poor birth outcomes among this group of infants.

Paternal factors may influence birth outcomes through a number of pathways that act directly [98] and indirectly through maternal factors [28, 39]. For our study we focused on paternal and maternal race as indicators of the social mechanisms that not only create disparities in environmental exposures [45], but also psychosocial stress associated with racial discrimination and systemic racism which are likely contributing to racial disparities in birth outcomes [29, 32, 40, 99]. The structural factors associated with systemic racism can also influence relationships within families. For example, non-Hispanic Blacks [100–102], Hispanics [103] and Asians [103, 104] are more likely to report higher rates of perceived racial/ethnic discrimination than non-Hispanic Whites, with men reporting higher rates than woman. Additionally, partnering with a man different from a woman’s race/ethnicity may lead to stress because of discriminatory treatment, family disapproval and reduced social support [105]. Thus, it is possible that experiences of everyday racism that mothers and/or their partners face may increase the stress experiences of a woman during pregnancy. Higher levels of stress have been associated with risk of preterm term birth and other adverse outcomes [106–111].

Another pathway could be through the effect of fathers on maternal health behaviors that include use of prenatal care, maternal smoking and other behaviors linked to adverse birth outcomes [39, 112, 113]. For example, one study of women whose residential partners were involved in their pregnancy were significantly more likely to receive prenatal care in the first trimester (OR = 1.42, 1.01, 1.99), and, among women who smoked at conception, those whose partners were involved in their pregnancy reduced their cigarette consumption 36% more than women whose partners were not involved [113]. In addition, paternal involvement has been shown to be critical for infant health as it is linked to financial and emotional support for their families [96]. It is also plausible that paternal involvement decreases stress.

Studies that have examined indicators of paternal involvement, such as marital status or completeness of paternal information on the birth certificate, have noted higher rates of preterm birth and low birth weights among father-absent births [31, 96, 97]. For married mothers, it is assumed that fathers are involved. However, marital status is not wholly indicative of paternal involvement. In our study, 93.5% of unmarried women were missing father’s information and yet 6.5% of married mothers had missing paternal information (Additional File 1). The highest rate of missing paternal information was among Black mothers in our study (36%). The second highest was among AIAN mothers at 20%. The rate of missing paternal information among AAPI and White mothers was 4% and 8% respectively. The reason why so many fathers are unreported on birth certificates is unclear [31, 95]. In addition to absence of a father during pregnancy as a reason for missing paternal data on birth certificates, other speculations include medical emergency admissions, administrative errors by attending physician or other health care staff (intentional or not) or financial and institutional barriers (e.g., state eligibility requirements for public assistance) that hinder the acknowledgment of the father on the birth certificate or involvement during pregnancy [114, 115]. In our study we found strong association between father’s “race not stated” (an indicator of incomplete paternal information on birth certificate) and birthweight, as well as evidence of effect modification by father’s “race not stated” on PM_2.5_ – birthweight associations, controlling for smoking and adequacy of prenatal care. This result confirms findings by Gould and colleagues who argued that incomplete birth certificates provide an important maker for identifying high-risk women and vulnerable infants [116]. From our perspective, dads’ missing information on birth certificates could be an indicator of macro-levels processes that lead to not only variability in exposure to air pollution, but also act synergistically with pollutant exposures negatively impacting birth outcomes. More attention is needed on the “determinants of the determinants,” the macro-level processes and social structures that “disproportionately place certain families in harmful contexts to begin with” [117].

The results of this study point to several avenues for future research. Our findings, coupled with those of others, suggest a need to consider socio-ecological contexts of both moms and dads individually and together in their relationship with each other in studies of environmental pollutant impact on birth outcomes. Collecting individual level information directly from fathers on their involvement during pregnancy and afterwards and their relationships to the children’s mothers is challenging [118]. The ECLS-B is one of the first nationally representative studies of children in the U.S. to collect information directly from fathers. In the ECLS-B at the 9-month baseline assessment and during follow-up waves, children’s resident and nonresident fathers completed short self-administered questionnaires responding to questions about themselves, their attitudes about fatherhood, and their involvement with their children [49, 118]. Yet, the ECLS-B struggled to recruit men, as 25% of eligible resident fathers and 50% of eligible nonresident fathers were not included in the 9-month, baseline data[119]. At nine months of age, the study baseline, 79% of the infants in the ECLS-B lived with their biological fathers [118]. Of those children who did not, 21% of these children had fathers who did not meet the study’s contact requirement [118]. In our ECLS-B study sample, 81% were living with both biological parents at nine months. Among those with missing paternal information on birth certificates, 68% were living in household without fathers (Additional File 1). Few prior studies have examined links between birth outcomes, childbearing intention and data from resident fathers among the ECLS-B participants [113, 120–123]. As follow-up to our current study, we plan to investigate the limited father questionnaire data regarding prenatal/neonatal experiences, particularly from non-resident fathers, to begin to disentangle the multiple social stressors for which paternal information may be a proxy and are relevant to our findings. However, we note this individual level information may not help answer our larger question about macro level precursors of inhibitory contexts (e.g., poverty, limited access to resources) that lead to unequal environmental exposures and missing paternal information on birth certificates that appears to enhance the negative effects of air pollution on birth weight. We argue this may be an excellent avenue for future interdisciplinary collaborations between environmental health scientists, reproductive health scientists, sociologists and family science researchers.

Our study is not without limitations. Similar to previous air pollution and birth outcomes studies, non-differential exposure misclassification is one potential problem which could bias our results towards null findings, although we are uncertain if this is the case[124]. We assumed ambient air pollutant concentrations at zip code levels were reasonable proxies of individual exposures. The restricted ECLS-B dataset only provides zip code for study participants. Therefore, our analysis is limited to using area-level exposures instead of personal exposures or ambient concentrations at more refined geographic levels (e.g., census tract or actual home address), which may have reduced variability in air pollution exposure at the individual level. However, it has been recommended to use area-based exposure measures instead of personal exposures as a means to avoid confounding by personal behavior differences [125]. Further, area-based air pollution measures have been used in previous large-scale of air pollution and birth outcomes studies [24, 51, 75, 126]. As in these previous studies, we could not account for residential mobility during pregnancy, the impact of which on effect sizes has been deemed modest to negligible, especially if using area-based measure of air pollution [127–130]. In addition to being used by previously published studies, area level measures offer the advantage of being more informative for policy change. State and Federal air pollution regulations are generally focused on ambient levels of air pollution. Data quality of birth records is another limitation, which is a common for all air pollution and birth outcome studies. Some health items on the birth certificate are known to be under-reported[131, 132], which may also limit the explanatory power of our analyses. Despite efforts to standardize the US birth certificate, data quality remains an issue as it is up to each state to implement improvements [131]. Another limitation is modeling the association between each trimester exposure and outcome separately if identification of a specific window of susceptibility is desired. Using trimester average exposures to estimate effects without controlling for exposure in other trimesters is a common approach and was used by earlier studies that specifically looked at effect modification by race/ethnicity [24, 62, 72, 75]. However, researchers using a simulation study have shown that separate trimester models may bias results, particularly with respect to the ranking of the trimester-specific effect sizes [133]. For many of the covariates used in our analyses, we relied on self-report from the ECLS-B questionnaires and parent interviews. There also may be concerns related to residual confounding. Many socioeconomic and sociodemographic characteristics are strong predictors of birth outcomes (birth weight) and air pollution. We adjusted our pollutant models for, maternal marital status, maternal educational attainment and poverty. We cannot, however, exclude a possible influence of other confounders. Another potential problem is mismatch of individual zip codes between ECLS-B dataset and the U.S. EPA air pollution data. However, we found that the rate of missing air pollution data was less than 10%. Additionally, we were not able to consider other pollutants or the constituents of PM2.5 in the analysis.

Our study has several strengths. This is the first national assessment of effect modification by both maternal and paternal race in the relationship between PM_2.5_ and birthweight. Previous studies assessing effect modification of race on the relationship between PM_2.5_ and birth outcomes have mostly been conducted in California, northeastern states, individual states and/or single urban areas with varying levels of population diversity. Studies of air pollution effects on birth outcomes have rarely considered the impact of paternal factors or on the racialized differences therein. Instead of treating paternal race as missing we included “race not stated” as its own category. Previous studies have shown that paternal race/ethnicity influences birth outcomes and that “missing” or “not stated” paternal race/ethnicity may be an important risk factor. Using the ECLS- data set, we were able to leverage demographic information from the 9-month records (e.g., residential zip code, urbanicity, poverty and US region). Additionally, we adjusted for maternal smoking, which has been identified as a serious limitation of previous studies where this risk factor was not taken into consideration. The PM_2.5_ measures were derived from U.S. EPA’s CMAQ model, which is a widely used and validated model providing data for the entire country. Use of CMAQ allowed us to assign air pollution exposures to urban and rural ECLS-B participants (where air monitors are sparse).

## Conclusion

In summary, several prior studies have examined differential effect of prenatal PM_2.5_ exposures on birth weight by race, though ours is the first using a nationally representative sample of births which included parental race/ethnicity information. We found disparities in prenatal PM_2.5_ exposures by both maternal and paternal race. Both maternal and paternal race/ethnicity were independently associated with birth outcomes in non-pollutant models. We found prenatal PM_2.5_ was associated with reduced birthweights during second and third trimester and over the entire gestational period in adjusted models, although results did not reach statistical significance. However, in models stratified by maternal race and paternal race, PM_2.5_ was statistically significantly associated with lower birthweights among AAPI mothers and fathers and among births where father’s race was not reported. These data suggest that paternal characteristics should be used, in addition to maternal characteristics, to describe the risks of adverse birth outcomes and that missing paternal information on birth certificate is important indicator of harmful environmental pollutant exposures and higher/enhanced risk.

## Supplementary Information

**Additional file 1: Supplementary Table 1. **Comparison of results from U.S. based studies that examined effect modification by parental race. **Supplementary Table 2. **Characteristics of U.S. singleton term births born in 2001 by birth weight status, Early Childhood Longitudinal Study-Birth Cohort (ECLS-B) study sample. **Supplementary Table3. **Cross-tabulation of maternal and paternal race/ethnicity for U.S. singleton term births born in 2001, Early Childhood Longitudinal Study-Birth Cohort (ECLS-B) study sample. **Supplementary Table 4. **Maternal marital status by maternal and paternal race/ethnicity for U.S. singleton term births born in 2001, Early Childhood Longitudinal Study-Birth Cohort (ECLS-B) study sample. **Supplementary Table 5. **Household poverty status by maternal and paternal race/ethnicity and type of father at 9-months for U.S. singleton term births born in 2001, Early Childhood Longitudinal Study-Birth Cohort (ECLS-B) study sample. **Supplementary Table 6. **Type of father at 9-months by maternal race/ethnicity for U.S. singleton term births born in 2001, Early Childhood Longitudinal Study-Birth Cohort (ECLS-B) study sample. **Supplementary Table 7. **Type of father at 9-months by paternal race/ethnicity for U.S. singleton term births born in 2001, Early Childhood Longitudinal Study-Birth Cohort (ECLS-B) study sample. **Supplementary Table 8. **Maternal educational attainment by paternal race/ethnicity for U.S. singleton term births born in 2001, Early Childhood Longitudinal Study-Birth Cohort (ECLS-B) study sample. **Supplementary Table 9. **Maternal educational attainment by maternal race/ethnicity for U.S. singleton term births born in 2001, Early Childhood Longitudinal Study-Birth Cohort (ECLS-B) study sample. **Supplementary Table 10. **Maternal educational attainment by type of father at 9-months for U.S. singleton term births born in 2001, Early Childhood Longitudinal Study-Birth Cohort (ECLS-B) study sample. **Supplementary Table 11. **Summary of average daily PM2.5 levels during each trimester and whole pregnancy. **Supplementary Table 12. **Correlation of PM2.5 across trimesters. **Supplementary Table 13. **Crude and adjusted odds ratios of term low birth weight (LBW) associated with selected non-pollutant variables among the ECLS-B study population. **Supplementary Table 14. **Crude and adjusted odds ratios of term low birth weight (LBW) associated with average PM2.5 exposures by trimester and whole pregnancy among the ECLS-B study population. **Supplementary Table 15. **Adjusted odds ratios of term low birth weight (LBW) associated with average PM2.5 exposures by trimester and whole pregnancy among the ECLS-B study population stratified by maternal and paternal race.

## Data Availability

The data that support the findings of this study are available from the U.S. Department of Education, National Center for Education Statistics but restrictions apply to the availability of these data, which were used under license for the current study, and so are not publicly available.
