# Supplementary material for: Both parents matter: a national-scale analysis of parental race/ethnicity, disparities in prenatal PM2.5 exposures and related impacts on birth outcomes
Source: Environ Health. 2022 May 6;21:47. doi: 10.1186/s12940-022-00856-w (PMC9074320; doi:10.1186/s12940-022-00856-w)
Supplement: Supplementary file 1 — Additional file 1: Supplementary Table 1. Comparison of results from U.S. based studies that examined effect modification by parental race. Supplementary Table 2. Characteristics of U.S. singleton term births born in 2001 by birth weight status, Early Childhood Longitudinal Study-Birth Cohort (ECLS-B) study sample. Supplementary Table3. Cross-tabulation of maternal and paternal race/ethnicity for U.S. singleton term births born in 2001, Early Childhood Longitudinal Study-Birth Cohort (ECLS-B) study sample. Supplementary Table 4. Maternal marital status by maternal and paternal race/ethnicity for U.S. singleton term births born in 2001, Early Childhood Longitudinal Study-Birth Cohort (ECLS-B) study sample. Supplementary Table 5. Household poverty status by maternal and paternal race/ethnicity and type of father at 9-months for U.S. singleton term births born in 2001, Early Childhood Longitudinal Study-Birth Cohort (ECLS-B) study sample. Supplementary Table 6. Type of father at 9-months by maternal race/ethnicity for U.S. singleton term births born in 2001, Early Childhood Longitudinal Study-Birth Cohort (ECLS-B) study sample. Supplementary Table 7. Type of father at 9-months by paternal race/ethnicity for U.S. singleton term births born in 2001, Early Childhood Longitudinal Study-Birth Cohort (ECLS-B) study sample. Supplementary Table 8. Maternal educational attainment by paternal race/ethnicity for U.S. singleton term births born in 2001, Early Childhood Longitudinal Study-Birth Cohort (ECLS-B) study sample. Supplementary Table 9. Maternal educational attainment by maternal race/ethnicity for U.S. singleton term births born in 2001, Early Childhood Longitudinal Study-Birth Cohort (ECLS-B) study sample. Supplementary Table 10. Maternal educational attainment by type of father at 9-months for U.S. singleton term births born in 2001, Early Childhood Longitudinal Study-Birth Cohort (ECLS-B) study sample. Supplementary Table 11. Summary of average daily PM2.5 levels duri [file 12940_2022_856_MOESM1_ESM.docx]

**Additional File 1**

**Supplementary Table 1. Comparison of results from U.S. based studies that examined effect modification by parental race**

**Supplementary Table 2. Characteristics of U.S. singleton term births born in 2001 by birth weight status, Early Childhood Longitudinal Study-Birth Cohort (ECLS-B) study sample**

**Supplementary Table3. Cross-tabulation of maternal and paternal race/ethnicity for U.S. singleton term births born in 2001, Early Childhood Longitudinal Study-Birth Cohort (ECLS-B) study sample**

**Supplementary Table 4. Maternal marital status by maternal and paternal race/ethnicity for U.S. singleton term births born in 2001, Early Childhood Longitudinal Study-Birth Cohort (ECLS-B) study sample**

**Supplementary Table 5. Household poverty status by maternal and paternal race/ethnicity and type of father at 9-months for U.S. singleton term births born in 2001, Early Childhood Longitudinal Study-Birth Cohort (ECLS-B) study sample**

**Supplementary Table 6. Type of father at 9-months by maternal race/ethnicity for U.S. singleton term births born in 2001, Early Childhood Longitudinal Study-Birth Cohort (ECLS-B) study sample**

**Supplementary Table 7. Type of father at 9-months by paternal race/ethnicity for U.S. singleton term births born in 2001, Early Childhood Longitudinal Study-Birth Cohort (ECLS-B) study sample**

**Supplementary Table 8. Maternal educational attainment by paternal race/ethnicity for U.S. singleton term births born in 2001, Early Childhood Longitudinal Study-Birth Cohort (ECLS-B) study sample**

**Supplementary Table 9. Maternal educational attainment by maternal race/ethnicity for U.S. singleton term births born in 2001, Early Childhood Longitudinal Study-Birth Cohort (ECLS-B) study sample**

**Supplementary Table 10. Maternal educational attainment by type of father at 9-months for U.S. singleton term births born in 2001, Early Childhood Longitudinal Study-Birth Cohort (ECLS-B) study sample**

**Supplementary Table 11 . Summary of average daily PM2.5 levels during each trimester and whole pregnancy**

**Supplementary Table 12. Correlation of PM2.5 across trimesters**

**Supplementary Table 13 . Crude and adjusted odds ratios of term low birth weight (LBW) associated with selected non-pollutant variables among the ECLS-B study population**

**Supplementary Table 14 . Crude and adjusted odds ratios of term low birth weight (LBW) associated with average PM2.5 exposures by trimester and whole pregnancy among the ECLS-B study population**

**Supplementary Table 15. Adjusted odds ratios of term low birth weight (LBW) associated with average PM2.5 exposures by trimester and whole pregnancy among the ECLS-B study population stratified by maternal and paternal race**

**Supplementary Table 1. Comparison of results from U.S. based studies that examined effect modification by parental race**

| **Author** | **Study Location (Time Period)** | **Number**  **of Births** | **Outcome** | **PM2.5 Exposure Metric** | **Approach** | **EM and/or Interaction Results** | **Smoking adjusted** | **Confounders considered** |
| --- | --- | --- | --- | --- | --- | --- | --- | --- |
| Basu et al. 2004 | California (2000) | 16,693 | CBW | county level, 5-mile and 1-mile radii averages based on monitoring stations | Effect Modification/ Stratification | County-level data produced a stronger negative association than the neighborhood-monitored air pollutant data for both the non-Hispanic white [5 mile: β for CBW (g) per 1 mg/m3 increase in PM2.5 = -1.52 (95% confidence interval: -3.52, 0.48), County: β= -4.04 ( -6.71, -1.37)] and Hispanic sample populations [5 mile: β= -2.49 ( -4.53, -0.45), County: β = -4.35 ( -7.47, -1.23)]. The estimates found for the Hispanic population suggest a slightly stronger association between PM2.5 and birth weight compared to those found for the non-Hispanic white population. This difference, however, was not statistically significant. However, using data from monitors within 1 mile of the mother’s residence, stronger associations were found for the non-Hispanic white population compared to the Hispanic population. | N | 1, 5-7, 9 |
| Basu et al. 2014 | California  (2000 – 2006) | 646,296 | CBW | Zip code tabulation area level average based on monitoring stations within 20km | Effect Modification/ Stratification | Asians, Blacks, and Hispanics, compared to Whites, exhibited smaller birth weight reductions for most PM2.5 constituents. However, Black and Hispanic mothers had greater reductions in birth weight with increases in exposure to total PM2.5 mass compared to White and Asian mothers. CBW (g) per 1µg/m3 PM2.5 (95% CI) Hispanic: -0.74344 (-1.14668, -0.34019); White: -0.83862 (-1.41021, -0.26702); Asian: -1.14198 (-2.05802, -0.22594); Black: -1.05944 (-2.18066, 0.061783). | N | 2, 3, 6, 7, 9, 17, 18, 25 |
| Bell et al. 2007 | Connecticut and Massachusetts (1999 – 2002) | 358,504 | CBW | county level average based on monitoring stations | Interaction | Black mothers at greater risk. CBW (g) per IQR of PM2.5: -22.6 (-29.3, -15.9) for Black mothers; -14.7 (-17.3, -12.0) for white mothers | Y | 1-7, 9, 11, 13, 17, 19 |
| Bell et al. 2010 | Connecticut and Massachusetts (1999 – 2000) | 76,788 | CBW | county level average based on monitoring stations | Interaction | For all pollutants, a given increment in exposure had larger associations among infants of African American mothers than those of white mothers (approximately, 35%–100% higher). However, these differences by race did not reach statistical significance for any given pollutant. CBW (g) per IQR increase in PM2.5 constituents (95% CI) : Zinc −7 (−13 , −2) for white mothers, −10 (−22, 3) for African American mothers; Elemental carbon −6 (−13, 1) for Whites, −9 (−24 to 7) for African Americans; Road dust −5 (−11, 1) for Whites, −7 (−22, 7) for African Americans; Silicon −5 (−10 to 0) for whites , −9 (−22 to 3) for African Americans; Aluminum −5 (−11, 0) for Whites, −9 (−22, 5) for African Americans; Vanadium −4 (−8, 0) for Whites, −9 (−18, 1) for African Americans; Nickel −6 (−12, −1) for whites , −12 (−24, 0) for African Americans. | Y | 1, 2, 3, 4, 6, 7, 9, 17, 19, 11, 13, 31 |
| Bell et al. 2012 | Connecticut and Massachusetts (2000 – 2004) | 76,788 | CBW and LBW | county level average based on monitoring stations | Interaction | Effect estimates for infants of African–American mothers were higher than those of white mothers, although the confidence intervals overlapped. CBW (g) per IQR increase in PM2.5-Potassium(95% CI) : -7.68 (-12.2, -3.16) for all births; -7.22 (-12.1, -2.33) for White mothers; -13.5 (-24.8, -2.08) for African American mothers. CBW (g) per IQR increase in PM2.5-Titanium (95% CI) for:-6.64 (-11.8, -1.46) for all births; -6.64 (-12.4, -0.93) for white mothers; -10.8 (-24.6, 3.00) for African American mothers. Percent change in risk of low birth weight (95% CI) per IQR increase in PM2.5-Potassium: 8.75% (1.24, 16.8%) for all births; 7.55% (-0.92,16.8%) for white mothers; 11.7% (-2.57, 28.0%) for African American . Percent change in risk of low birth weight (95% CI) per IQR increase in PM2.5-Titanium: 12.1% (3.55, 21.4%) for all births; 10.0% (0.15, 20.9%) for White mothers; 23.7% (5.29, 45.4%) for African American mothers. | Y | 1, 2, 3, 4, 5, 6, 7, 9, 11, 13, 19, 31 |
| Choi et al. 2012 | New York City (1998 – 2005) | 615 | CBW | personal air monitoring for PAHs during 3rd trimester (a component of PM) | Interaction | A 25th—80th percentile increase in airborne PAH exposure was associated with a -164 g greater birth weight reduction for the newborns of African-American women (95% CI —51 to —277 g; p<0.01) than the newborns of the Dominican women. | Y | 1, 2, 3, 9, 14, 15, 18, 27 |
| Darrow et al. 2011 | Atlanta, Georgia (1994 – 2004) | 406,627 | CBW | county level average based on monitoring stations | Interaction | The pollution × race interaction terms were statistically significant for PM2.5 and other pollutants studied. Overall, authors observed more statistically significant associations between air pollutants and birth weight in the Hispanic and non- Hispanic black groups than in the non-Hispanic white group. CBW (g) per IQR of PM2.5 during 3rd trimester: 0.1 (–6.0 to 6.2) for White mothers; –6.3 (–12.5 to –0.2) for Black mothers; –10.2 (–17.6 to –2.7) for Hispanic mothers. | Y | 1, 2, 3, 5, 6, 9, 11, 17, 18 |
| Ebisu and Bell 2012 | Connecticut, Maryland, Massachusetts, Delaware, New Hampshire, New Jersey, New York, Pennsylvania, Rhode Island, Washington DC, Vermont, Virginia, West Virginia  (2000 – 2007) | 1,207,800 | LBW | county level average based on monitoring stations | Interaction | Data not shown. Relative risk of LBW associated with IQR increase in PM2.5 elemental carbon was 7.3% (95%CI: 4.9, 9.6%) ***lower*** among African American mothers compared with white mothers. | Y | 1,2, 3, 4, 5, 6, 7, 9, 11, 13, 17, 18, 19, 31, 32 |
| Enders et al. 2019 | California  (2003 – 2013) | 2,719,596 | LBW | Zip code tabulation area level average based on monitoring stations within 20km | Effect Modification/ Stratification | Data not shown. Stratified by maternal race, none of the quartiles of PM2.5 were associated with an increased risk of term LBW. But the authors note that their stratified analyses often showed that in sub-populations where ORs for PM10–2.5 were strong, ORs for PM2.5 exposure were weak, and vice versa. For example they observed a strong association between PM10–2.5 and TLBW among Non-Hispanic Black mothers, but did not see an association between PM2.5 and TLBW in this group | Y | 2, 4, 6, 7, 8, 9, 10, 11, 16, 17, 18, 19, 25, 34, |
| Fong et al. 2019 | Massachusetts  (2001 – 2013) | 775,768 | CBW | air pollutant modeling estimating exposure at maternal residential address with 1kmx 1km resolution | Interaction | Data not shown. Found no evidence of effect modification by census block proportion of Black population. Speculated that this lack of finding due to differences in study population since only include births in Massachusetts. | Y | 1, 2, 3, 4, 5,6, 7, 9, 11, 21, 25, 27 |
| Geer et al. 2012 | Texas  (1998 – 2004) | 1,548,904 | CBW | county level average based on monitoring stations | Interaction | ***Higher*** birth weight was associated with ***increase in gestational exposure*** to PM2.5 in Hispanic mothers. CBW (g) per IQR of PM2.5: 1.93 (-3.95, 7.82) for Black mothers; 3.35 (1.37, 5.33) for Hispanic mothers, -3.44 (-7.01, 0.12) for Whites | Y | 1, 2, 3, 4, 5, 6, 7, 9, 11, 13, 33 |
| Laurent et al. 2014 | Los Angeles County, California  (2001 – 2008) | 960,945 | LBW | air pollutant modeling estimating exposure at maternal residential address with 4kmx 4km resolution | Effect Modification/ Stratification | Authors stated that for analyses stratified by maternal race/ethnicity, most significant associations between LBW and primary PM were observed in infants of Hispanic mothers. From Table A.4, LBW Adjusted ORs associated with IQR increase in PM2.5 (95%CI) 0.962 (0.931,0.994) for Asian mothers; 1.039 (1.002, 1.076) for African American mothers; 1.028 (1.014, 1.041) for Hispanic mothers; 1.036 (1.011, 1.061) for White mothers; 0.86 (0.79 0.93) for mothers of other racial/ethnicity groups | N | 1, 2, 3, 4, 7, 9, 25 |
| Morello-Frosch et al. 2010 | California  (1996 – 2006) | 3,545,177 | CBW | Zip code tabulation area level average based on monitoring stations within 10km | Effect Modification/ Stratification | PM2.5 effect estimates for decreases in average BW were strongest for African Americans. From Figure 4 CBW (g) per unit of 10 µg/m^3^ PM2.5: -24 for Black mothers; -15 for white mothers; -10 for Hispanics; -14 for Asian/Pacific Islanders | N | 1, 2, 3, 4, 5, 6, 7, 9, 17, 18, 25, 27 |
| Ng et al. 2017 | California  (2002 – 2009) | 1,050,330 | LBW | Zip code tabulation area level average based on monitoring stations within 20km | Effect Modification/ Stratification | Associations between PM2.5 total mass and sources and term LBW varied by race/ethnicity, with the greatest risk of term LBW found in blacks, followed by Hispanics. Lower risk of term LBW was associated with PM2.5 total mass for Asian mothers. From Figure 2, adjusted % change in odds LBW per IQR increase in total PM2.5, 12 % for Black mothers, 5% for Whites, 6% for Hispanics, and -7% for Asians. | N | 2, 6, 7, 9, 17, 19, 25, |
| Parker et al. 2005 | California  (2000) | 18,247 | CBW | Average of monitors within 5 miles of maternal residence | Interaction | Interactions between PM 2.5 and maternal race/ethnicity and maternal education were not statistically significant (data not shown) and thus not retained in their models. | N | 1,5-7, 9, 11, 18 - 21, 25 |
| Parker and Woodruff 2008 | USA by region (2001 – 2003) | 401,273 | CBW | county level average based on monitoring stations | Interaction | Data not shown. Significant association between PM2.5 and race/ethnicity with **all associations positive**. ***More pollution associated with higher birth weights*** | N | 1, 6, 9,17, 18, 25, 26 |
| Perera et al. 2003 | New York City (1998 – 2002) | 263 | CBW | personal air monitoring for PAHs during 3rd trimester (a component of PM) | Interaction and Effect Modification/ Stratification | PAHs had a significant adverse effect on birth weight among African-American but not Dominican infants even after adjusting for co-exposure to chlorpyrifos. % change in BW per unit increase in air PAH ng/m^3^ : -10% (p-value =0.02) for African American mothers and 0.90 % (p-value =0.81) for Dominican mothers | Y | 1, 2, 3, 9, 11, 27, |
| Rhee et al. 2019 | Boston, Massachusetts (2007 - 2015) | 3,366 | CBW | air pollutant modeling estimating exposure at maternal residential address with 1kmx 1km resolution | Effect Modification/ Stratification | The effect of PM2.5 during the 2nd trimester on reduced birthweight was stronger among non-Hispanic Black mothers and was marginally significant among immigrants . From Figure 3, mean CBW (g) per 1µg/m^3^ of PM2.5 during second trimester: 14.0 (−20.1, 48.1) for White mothers; −15.2 (-29.4, −0.9) for Black mothers; −8.8 (−27.5, 10.0) for Hispanic mothers; 23.1 (−42.5, 88.6) for other race/ethnicity; −14.4 (−31.7, 2.9) for immigrant mothers. | Y | 3, 6, 7, 9, 11, 18, 25, 27 |
| Salihu et al. 2012 | Hillsborough County, Florida (2000 – 2007) | 103,961 | LBW | Zip code area level average based on monitoring stations, then mothers exposures were dichotomized, exposed if above median concentration | Interaction and Effect Modification/ Stratification | Compared to infants born to white mothers, Blacks exposed to any PM had the greatest odds for all outcomes studied with LBW Adjusted OR = 2.58 (95%CI 2.33, 2.85), VLBW Adjusted OR = 3.32 (95% CI 2.56, 4.30). Hispanic women LBW Adjusted OR = 1.36 (95%CI 1.21, 1.52), VLBW Adjusted OR = 1.44 (95% CI 1.07, 1.94). Other Race/ethnicity LBW Adjusted OR = 1.60 (95%CI 1.42, 1.79), VLBW Adjusted OR = 1.73 (95% CI 1.27, 2.35). | Y | 1, 3, 4, 5, 7,9, 17, 21, 27 |

Confounders may be addressed by inclusion of a variable in models or restriction

Confounders: 1 = parity, 2 = gestational length and or age (addressed if limited to term births), 3 = sex of infant, 4 = prenatal care, 5= maternal marital status, 6 = maternal age, 7 = maternal socioeconomic status such as education, or household income , 8 = paternal education, 9 = maternal race, 10= paternal race, 11 = maternal tobacco use, 12 = maternal drug use, 13 = maternal alcohol use, 14 = maternal exposure to secondhand smoke, 15 = maternal weight gain, 16 = maternal pre-pregnancy weight, 17 = year of birth, 18 = season of birth, 19 = weather, 20 = time since last birth, 21 = gestational diabetes, 22 = previous low birth weight delivery, 23 = maternal working status, 24 = toxemia, 25 = community level ses characteristics, 26 = urban or rural, 27 = other complications/risk factors like anemia, uterine bleeding, 28 = previous terminations, 29 = restricted to no maternal hypertension, uterine bleeding or diabetes, 30 = commute time, percent time working, 31 = delivery method; 32 = state of residence, 33 =public health administrative region, 34 = paternal age

**Supplementary Table 2. Characteristics of** **U.S. singleton term births born in 2001 by birth weight status, Early Childhood Longitudinal Study-Birth Cohort (ECLS-B) study sample**

|  | Normal  Birth  Weight | Low  Birth  Weight^b^ |
| --- | --- | --- |
| Child Sex |  |  |
| Male | 51% | 40% |
| Female | 49% | 60% |
| Parity |  |  |
| First child | 40% | 50% |
| Not first child | 60% | 50% |
| Adequacy of prenatal care (Kessner Index) | | |
| Adequate | 78% | 74% |
| Intermediate | 17% | 21% |
| Inadequate | 5% | 5% |
| Maternal education |  |  |
| < 12 years | 20% | 28% |
| 12 years | 31% | 37% |
| 13 - 15 years | 22% | 20% |
| > 15 years | 25% | 15% |
| unknown | 1% | 1% |
| Maternal race/ethnicity | | |
| White, non-Hispanic | 62% | 48% |
| Black, non-Hispanic | 14% | 31% |
| Hispanic, any race | 21% | 17% |
| AAPI^a^ | 3% | 4% |
| American Indian/Alaskan Native | 0.7% | 0.90% |
| Paternal race/ethnicity | | |
| White, non-Hispanic | 55% | 42% |
| Black, non-Hispanic | 10% | 21% |
| Hispanic, any race | 19% | 15% |
| AAPI^a^ | 3% | 3% |
| American Indian/Alaskan Native | 0.6% | 0.50% |
| Not stated | 12% | 18% |
| Maternal marital status | | |
| Married | 69% | 52% |
| Not Married | 31% | 48% |
| Mother's age |  |  |
| < 20 yrs | 10% | 17% |
| 20 – 24 | 25% | 31% |
| 25 - 29 | 27% | 22% |
| 30 - 34 | 24% | 18% |
| 35 - 39 | 11% | 11% |
| > 39 | 2% | 2% |
| Paternal age |  |  |
| < 45 yrs | 86% | 79% |
| ≥ 45 | 2% | 3% |
| Not stated | 11% | 18% |
| Urbanicity |  |  |
| Rural | 14% | 16% |
| Urban | 86% | 83% |
| Region |  |  |
| Northeast | 17% | 14% |
| Midwest | 23% | 20% |
| South | 37% | 42% |
| West | 22% | 25% |
| Smoked during pregnancy | | |
| Yes | 10% | 22% |
| No | 76% | 65% |
| Unknown | 14% | 14% |
| Poverty |  |  |
| <185% Poverty | 46% | 62% |
| ≥ 185% | 54% | 38% |

Notes: Percentages are weighted to take the complex sampling design into account. ^a^Asian American and Pacific Islander (AAPI), includes Chinese, Japanese, Hawaiian, Filipino, Asian Indian, Korean, Samoan, Vietnamese, Guamanian and other Asian or Pacific Islander. ^b^Low birth weight defined as <2500 g.

**Supplementary Table 3. Cross-tabulation of maternal and paternal race/ethnicity for U.S. singleton term births born in 2001, Early Childhood Longitudinal Study-Birth Cohort (ECLS-B) study sample**

|  |  | Paternal Race/Ethnicity | | | | |
| --- | --- | --- | --- | --- | --- | --- |
| Maternal Race/Ethnicity | **White** | **African American** | **Hispanic** | **AAPI^a^** | **American Indian/Alaskan Native** | **Not Stated** |
| **White** | 85% | 2% | 4% | 3% | 6% | 9% |
| **African American** | 3% | 60% | 2% | 1% | 1% | 36% |
| **Hispanic** | 8% | 2% | 81% | 3% | 1% | 10% |
| **AAPI**^a^ | 15% | 2% | 3% | 80% | 0% | 3% |
| **American Indian/Alaskan Native** | 36% | 5% | 8% | 1% | 30% | 20% |

Notes: F statistic = 1018.00, p-value= 0.000. Percentages are weighted to take the complex sampling design into account. ^a^Asian American and Pacific Islander (AAPI), includes Chinese, Japanese, Hawaiian, Filipino, Asian Indian, Korean, Samoan, Vietnamese, Guamanian and other Asian or Pacific Islander. Table should be read across rows (i.e., 85% of births to White mothers reported White fathers on birth certificate).

**Supplementary Table 4. Maternal** **marital status by maternal and paternal race/ethnicity for U.S. singleton term births born in 2001, Early Childhood Longitudinal Study-Birth Cohort (ECLS-B) study sample**

|  | Married | Not Married |
| --- | --- | --- |
|  | % | % |
| Maternal race/ethnicity |  |  |
| White, non-Hispanic | 79% | 21% |
| Black, non-Hispanic | 33% | 67% |
| Hispanic, any race | 59% | 41% |
| AAPI^a^ | 87% | 13% |
| American Indian/Alaskan Native | 46% | 54% |
|  |  |  |
| Paternal race/ethnicity |  |  |
| White, non-Hispanic | 87% | 13% |
| Black, non-Hispanic | 45% | 55% |
| Hispanic, any race | 56% | 45% |
| AAPI^a^ | 91% | 9% |
| American Indian/Alaskan Native | 65% | 35% |
| Not stated | 7% | 95% |
|  |  |  |

Notes: Percentages are weighted to take the complex sampling design into account. ^a^Asian American and Pacific Islander (AAPI), includes Chinese, Japanese, Hawaiian, Filipino, Asian Indian, Korean, Samoan, Vietnamese, Guamanian and other Asian or Pacific Islander.

**Supplementary Table 5. Household poverty status by maternal and paternal race/ethnicity and type of father at 9-Months for U.S. singleton term births born in 2001, Early Childhood Longitudinal Study-Birth Cohort (ECLS-B) study sample**

|  | At or Above Poverty | Below Poverty |
| --- | --- | --- |
|  | % | % |
| Maternal race/ethnicity |  |  |
| White, non-Hispanic | 67% | 33% |
| Black, non-Hispanic | 28% | 72% |
| Hispanic, any race | 29% | 71% |
| AAPIa | 67% | 33% |
| American Indian/Alaskan Native | 35% | 65% |
|  |  |  |
| Paternal race/ethnicity |  |  |
| White, non-Hispanic | 72% | 28% |
| Black, non-Hispanic | 35% | 64% |
| Hispanic, any race | 30% | 70% |
| AAPI^a^ | 69% | 31% |
| American Indian/Alaskan Native | 40% | 60% |
| Not stated | 19% | 82% |
|  |  |  |
|  |  |  |
| Type of Father at 9-months |  |  |
| Birth Father | 61% | 39% |
| Adoptive Father | 44% | 56% |
| Step Father | 23% | 77% |
| Foster Father | 55% | 45% |
| Mother’s Partner | 33% | 67% |
| Non-Resident Father | 19% | 81% |

Notes: Percentages are weighted to take the complex sampling design into account. ^a^Asian American and Pacific Islander (AAPI), includes Chinese, Japanese, Hawaiian, Filipino, Asian Indian, Korean, Samoan, Vietnamese, Guamanian and other Asian or Pacific Islander. Household poverty status (below 185% poverty) was determined by ECLS-B using data on household income and household size obtained during the parent interview and the U.S. Bureau of the Census’ weighted poverty thresholds for 2001 for households with children.

**Supplementary Table 6. Type of father at 9-months by maternal race/ethnicity for U.S. singleton term births born in 2001, Early Childhood Longitudinal Study-Birth Cohort (ECLS-B) study sample**

|  | Type of Father at 9-months  % | | | | |  | |
| --- | --- | --- | --- | --- | --- | --- | --- |
| Maternal Race/Ethnicity | **Birth Father** | **Adoptive Father** | **Step Father** | **Foster Father** | **Mother’s Partner** | **Non-Resident Father** |  |
| **White, non-Hispanic** | 88% | 0.06% | 0.3% | 0.05% | 0.6% | 11% | |
| **Black, non-Hispanic** | 43% | 0% | 0.4% | 0.16% | 0.4% | 56% | |
| **Hispanic, any race** | 83% | 0% | 0.2% | 0% | 0.6% | 17% | |
| **AAPI^a^** | 91% | 0% | 0% | 0% | 0.3% | 8% | |
| **American Indian/Alaskan Native** | 75% | 0% | 0.2% | 0% | 1% | 24% | |

Notes: F statistic = 51.8, p-value= 0.000 Percentages are weighted to take the complex sampling design into account. ^a^Asian American and Pacific Islander (AAPI), includes Chinese, Japanese, Hawaiian, Filipino, Asian Indian, Korean, Samoan, Vietnamese, Guamanian and other Asian or Pacific Islander. Table should be read across rows (i.e., 88 % of infants with White mothers were residing with in households with their biological fathers.

**Supplementary Table 7. Type of father at 9-months by paternal race/ethnicity for U.S. singleton term births born in 2001, Early Childhood Longitudinal Study-Birth Cohort (ECLS-B) study sample**

|  | Type of Father at 9-months  % | | | | |  | |
| --- | --- | --- | --- | --- | --- | --- | --- |
| Paternal Race/Ethnicity | **Birth Father** | **Adoptive Father** | **Step Father** | **Foster Father** | **Mother’s Partner** | **Non-Resident Father** |  |
| **White, non-Hispanic** | 93% | 0.03% | 0.2% | 0.0% | 0.4% | 6% | |
| **Black, non-Hispanic** | 60% | 0% | 0% | 0.2% | 0.2% | 39% | |
| **Hispanic, any race** | 87% | 0% | 0.2% | 0% | 0.2% | 13% | |
| **AAPI^a^** | 95% | 0% | 0% | 0% | 0.09% | 5% | |
| **American Indian/Alaskan Native** | 80% | 0% | 0% | 0% | 0.7% | 20% | |
| **Not stated** | 27% | 0.2% | 1% | 0.2% | 3% | 68% | |

Notes: F statistic = 68.3, p-value= 0.000 Percentages are weighted to take the complex sampling design into account. ^a^Asian American and Pacific Islander (AAPI), includes Chinese, Japanese, Hawaiian, Filipino, Asian Indian, Korean, Samoan, Vietnamese, Guamanian and other Asian or Pacific Islander. Table should be read across rows (i.e., 93 % of infants with White fathers reported on birth certificates were residing with in households with their biological fathers).

**Supplementary Table 8. Maternal educational attainment by paternal race/ethnicity for U.S. singleton term births born in 2001, Early Childhood Longitudinal Study-Birth Cohort (ECLS-B) study sample**

|  | Maternal Educational Attainment  % | | | | |
| --- | --- | --- | --- | --- | --- |
| Paternal Race/Ethnicity | **<12 yrs** | **12 yrs** | **13 – 15 yrs** | **>15 yrs** | **Unknown** |
| **White, non-Hispanic** | 8% | 29% | 25% | 37% | 0.3% |
| **Black, non-Hispanic** | 19% | 40% | 24% | 15% | 2% |
| **Hispanic, any race** | 45% | 28% | 15% | 10% | 2% |
| **AAPI**^a^ | 9% | 21% | 20% | 49% | 1% |
| **American Indian/Alaskan Native** | 23% | 36% | 25% | 16% | 0.8% |
| **Not stated** | 40% | 37% | 14% | 5% | 3% |

Notes: F statistic = 70.7, p-value= 0.000 Percentages are weighted to take the complex sampling design into account. ^a^Asian American and Pacific Islander (AAPI), includes Chinese, Japanese, Hawaiian, Filipino, Asian Indian, Korean, Samoan, Vietnamese, Guamanian and other Asian or Pacific Islander. Table should be read across rows (i.e., 8% of births with White fathers reported on birth certificates were to mothers with less than high school education.

**Supplementary Table 9. Maternal educational attainment by maternal race/ethnicity for U.S. singleton term births born in 2001, Early Childhood Longitudinal Study-Birth Cohort (ECLS-B) study sample**

|  | Maternal Educational Attainment  % | | | | |
| --- | --- | --- | --- | --- | --- |
| Maternal Race/Ethnicity | **<12 yrs** | **12 yrs** | **13 – 15 yrs** | **>15 yrs** | **Unknown** |
| **White, non-Hispanic** | 11% | 29% | 25% | 34% | 0.6% |
| **Black, non-Hispanic** | 24% | 43% | 20% | 11% | 2% |
| **Hispanic, any race** | 48% | 27% | 14% | 10% | 2% |
| **AAPI**^a^ | 9% | 21% | 20% | 49% | 1% |
| **American Indian/Alaskan Native** | 29% | 35% | 25% | 11% | 1% |

Notes: F statistic = 72.2, p-value= 0.000 Percentages are weighted to take the complex sampling design into account. ^a^Asian American and Pacific Islander (AAPI), includes Chinese, Japanese, Hawaiian, Filipino, Asian Indian, Korean, Samoan, Vietnamese, Guamanian and other Asian or Pacific Islander. Table should be read across rows (i.e., 11% of births among White mothers had mothers with less than high school education.

**Supplementary Table 10. Maternal educational attainment by type of father at 9-months for U.S. singleton term births born in 2001, Early Childhood Longitudinal Study-Birth Cohort (ECLS-B) study sample**

| Maternal Educational Attainment  % (95% CI) | | | | | |
| --- | --- | --- | --- | --- | --- |
| Type of Father at 9-months | **<12 yrs** | **12 yrs** | **13 – 15 yrs** | **>15 yrs** | **Unknown** |
| **Birth Father** | 18% | 29% | 23% | 30% | 1% |
| **Adoptive Father** | 56% | 0% | 0% | 44% | 0% |
| **Step Father** | 49% | 15% | 19% | 16% | 0% |
| **Foster Father** | 3% | 97% | 0% | 0% | 0% |
| **Mother’s Partner** | 40% | 48% | 8% | 4% | 0% |
| **Non-Resident Father** | 35% | 41% | 16% | 6% | 2% |

Notes: F statistic = 16.2, p-value= 0.000 Percentages are weighted to take the complex sampling design into account. Table should be read across rows (i.e., 18 % of infants residing in households with their biological fathers had mothers with less than high school education.

**Supplementary Table 11. Summary of average daily PM2.5 levels during each trimester and whole pregnancy**

|  |  |  | **Percentiles** | | | | |
| --- | --- | --- | --- | --- | --- | --- | --- |
| **Trimester Period** | **Mean µg/m^3^ (range)** | **SD** | **10th** | **25th** | **50th** | **75th** | **90th** |
| 1 | 15.6 (5.1 - 71.0) | 5.0 | 10.1 | 12.7 | 15.0 | 17.8 | 21.7 |
| 2 | 14.8 (5.0 - 60.2) | 4.4 | 9.2 | 11.9 | 14.5 | 17.4 | 20.1 |
| 3 | 14.4 (4.6 - 36.4) | 4.5 | 9.0 | 11.3 | 14.1 | 16.9 | 20.0 |
| Whole | 14.9 (5.3 - 36.4) | 3.9 | 10.0 | 12.5 | 14.8 | 17.1 | 19.5 |

**Supplementary Table 12. Correlation of PM2.5 across trimesters**

|  | **1st Trimester** | **2nd Trimester** | **3rd Trimester** | **Whole**  **Pregnancy** |
| --- | --- | --- | --- | --- |
| **1st Trimester** | 1.00 |  |  |  |
| **2nd Trimester** | 0.465 | 1.00 |  |  |
| **3rd Trimester** | 0.365 | 0.532 | 1.00 |  |
| **Whole Pregnancy** | 0.587 | 0.752 | 0.945 | 1.00 |

All statistically significant p <0.05

**Supplementary Table 13 . Crude and adjusted odds ratios of term low birth weight (LBW) associated with selected non-pollutant variables among the ECLS-B study population**

|  |  | Model 1 | Model 2 | Model 3 | Model 4 |
| --- | --- | --- | --- | --- | --- |
|  | **Crude** | **Non-pollutants associations (No race/ethnicity)** | **Non-pollutants associations (Maternal race/ethnicity only)** | **Non-pollutants associations**  **(Paternal race/ethnicity only)** | **Non-pollutants associations (Maternal and paternal race/ethnicity)** |
|  | ORs of TLBW | ORs of TLBW | ORs of TLBW | ORs of TLBW | ORs of TLBW |
| Parameter | β Estimate (95% CI) | β Estimate (95% CI) | β Estimate (95% CI) | β Estimate (95% CI) | β Estimate (95% CI) |
| Sex |  |  |  |  |  |
| Male | 0.64 (0.51, 0.80) | 0.61 (0.48, 0.79) | 0.62 (0.48, 0.80) | 0.61 (0.47, 0.79) | 0.62 (0.48, 0.80) |
| Female | reference | reference | reference | reference | reference |
| Parity |  |  |  |  |  |
| First child | reference | reference | reference | reference | reference |
| Not first child | 0.67 (0.54, 0.84) | 0.56 (0.43, .73) | 0.54 (0.42, 0.71) | 0.55 (0.41, 0.72) | 0.54 (0.41, 0.71) |
| Adequacy of prenatal care (Kessner Index) |  |  |  |  |  |
| Adequate | reference | reference | reference | reference | reference |
| Intermediate | 1.28 (0.95, 1.72) | 1.07 (0.78, 1.47) | 1.03 (0.75, 1.42) | 1.04 (0.76, 1.44) | 1.03 (0.75, 1.42 |
| Inadequate | 1.12 (0.70, 1.76) | 0.85 (0.50, 1.43) | 0.82 (0.50, 1.34) | 0.83 (0.50, 1.39) | 0.60 (0.53, 0.69) |
| Gestational length (weeks) | 0.62 (0.55, 0.71) | 0.60 (0.53, 0.68) | 0.60 (0.53, 0.69) | 0.59 (0.52, 0.68) | 0.60 (0.53, 0.69) |
| Maternal education |  |  |  |  |  |
| < 12 years | reference | reference | reference | reference | reference |
| 12 years | 0.87 (0.65, 1.17) | 1.11 (0.81, 1.55) | 1.00 (0.71, 1.41) | 1.04 (0.74, 1.4) | 1.00 (0.71, 1.41) |
| 13 - 15 years | 0.67 (0.46, 0.97) | 1.05 (0.69, 1.61) | 0.94 (0.60, 1.46) | 0.96 (0.62, 1.49) | 0.93 (0.59, 1.45) |
| > 15 years | 0.42 (0.30, 0.59) | 0.77 (0.49, 1.22) | 0.71 (0.43, 1.17) | 0.71 (0.43, 1.15) | 0.70 (0.42, 1.15) |
| unknown | 0.87 (0.27, 2.80) | 1.51 (0.43, 5.27) | 1.34 (0.37, 4.80) | 1.41 (09, 5.05) | 1.40 (0.38, 5.00) |
|  |  |  |  |  |  |
| Maternal race/ethnicity |  |  |  |  |  |
| White | reference |  | reference |  | reference |
| Black | 2.93 (2.29, 3.75) |  | 2.71 (2.00, 3.73) |  | 2.32 (1.48, 3.64) |
| Hispanic | 1.04 (0.78, 1.39) |  | 0.98 (0.64, 1.49) |  | 1.05 (0.60, 1.84) |
| AAPI^a^ | 1.54 (1.04, 2.28) |  | 1.80 (1.14, 2.81) |  | 1.66 (0.87, 3.15) |
| American Indian/Alaskan Native | 1.84 (1.09, 3.13) |  | 1.33 (0.77, 2.34) |  | 1.38 (0.75, 2.56) |
| Paternal race/ethnicity |  |  |  |  |  |
| White | reference |  |  | reference | reference |
| Black | 2.77 (2.03, 3.77) |  |  | 2.46 (1.72, 3.54) | 1.25 (0.74, 2.14) |
| Hispanic | 1.05 (0.76, 1.45) |  |  | 0.91 (0.58, 1.43) | 0.90 (0.49, 1.64) |
| AAPI^a^ | 1.56 (1.04, 2.33) |  |  | 1.69 (1.07, 2.66) | 1.12 (0.59, 2.10) |
| American Indian/Alaskan Native | 1.24 (0.57, 2.73) |  |  | 0.96 (0.43, 2.16) | 0.90 (0.49,1.64) |
| Not stated | 2.08 (1.52, 2.83) |  |  | 1.00 (0.38, 2.68) | 0.74 (0.26, 2.13) |
| Maternal marital status |  |  |  |  |  |
| Married | reference | reference | reference | reference | reference |
| Not Married | 2.07 (1.67, 2.56) | 1.60 (1.18, 2.18) | 1.35 (0.99, 1.83) | 1.37 (0.99, 1.89) | 1.34 (0.97, 1.85) |
|  |  |  |  |  |  |
| Maternal age (years) |  |  |  |  |  |
| < 20 yrs | 2.09 (1.48, 2.96) | 0.90 (0.57, 1.39) | 0.89 (0.57, 1.37) | 0.90 (0.58, 1.42) | 0.87 (0.57, 1.38) |
| 20 - 24 | 1.58 (1.17, 2.13) | 0.87 (0.61, 1.25) | 0.86 (0.60, 1.24) | 0.85 (0.60, 1.24) | 0.86 (0.58, 1.24) |
| 25 - 29 | 1.03 (0.73, 1.48) | 0.87 (0.59, 1.26) | 0.85 (0.59, 1.24) | 0.85 (0.60, 1.23) | 0.85 (0.59, 1.24) |
| 30 - 34 | reference | reference | reference | reference | reference |
| 35 - 39 | 1.24 (0.82, 1.86) | 1.21 (0.76, 1.91) | 1.20 (0.76, 1.89) | 1.25 (0.80, 1.97) | 1.22 (0.78, 1.92) |
| > 39 | 1.20 (0.55, 2.62) | 1.42 (0.63, 3.18) | 1.31 (0.58, 3.00) | 1.31 (0.58, 3.00) | 1.30 (0.57, 2.97) |
|  |  |  |  |  |  |
| Paternal age (years) |  |  |  |  |  |
| < 45 yrs | reference | reference | reference | reference | reference |
| ≥ 45 | 1.32 (0.64, 2.74) | 1.28 (0.61, 2.67) | 1.13 (0.54, 2.35) | 1.15 (0.56, 2.36) | 1.12 (0.54, 2.32) |
| Not stated | 1.77 (1.32, 2.35) | 0.98 (0.69, 1.40) | 0.81 (0.56, 1.17) | 1.25 (0.48, 3.28) | 1.18 (0.43, 3.27) |
|  |  |  |  |  |  |
| Urbanicity |  |  |  |  |  |
| Rural | reference | reference | reference | reference | reference |
| Urban | 0.33 (0.59, 1.71) | 0.95 (0.66, 1.36) | 0.83 (0.57, 1.21) | 0.96 (0.57, 1.26) | 0.83 (0.57, 1.22) |
|  |  |  |  |  |  |
| Region |  |  |  |  |  |
| Northeast | reference | reference | reference | reference | reference |
| Midwest | 1.03 (0.64, 1.65) | 1.18 (0.75, 1.86) | 1.21 (0.80, 1.83) | 1.21 (0.80, 1.83) | 1.21 (0.80, 1.83) |
| South | 1.36 (0.90, 2.03) | 1.36 (0.93, 1.99) | 1.26 (0.88, 1.82) | 1.28 (0.91, 1.82) | 1.26 (0.88, 1.80) |
| West | 1.32 (0.85, 2.05) | 1.62 (1.02, 2.58) | 1.93 (1.24, 3.00) | 1.77 (1.15, 2.75) | 1.92 (1.23, 2.98) |
|  |  |  |  |  |  |
| Smoked during pregnancy from BC |  |  |  |  |  |
| Yes | 2.46 (1.85, 3.28) | 2.27 (1.65, 3.11) | 2.65 (1.88, 3.75) | 2.42 (1.75, 3.34) | 2.66 (1.88, 3.75) |
| Unknown |  |  |  |  |  |
| No | reference | reference | reference | reference | reference |
|  |  |  |  |  |  |
| Poverty |  |  |  |  |  |
| <185% Poverty | 1.89 (1.49, 2.4) | 1.42 (1.04, 1.91) | 1.26 (0.92, 1.73) | 1.32 (0.97, 1.80) | 1.27 (0.93, 1.73) |
| => 185% | reference | reference | reference | reference | reference |

Notes: All analyses are weighted to take the complex sampling design into account. ^a^Asian American and Pacific Islander (AAPI), includes Chinese, Japanese, Hawaiian, Filipino, Asian Indian, Korean, Samoan, Vietnamese, Guamanian and other Asian or Pacific Islander.

**Supplementary Table 14 . Crude and adjusted odds ratios of term low birth weight (LBW) associated with average PM2.5 exposures by trimester and whole pregnancy among the ECLS-B study population**

|  | Model 5 | Model 6 | Model 7 | Model 8 |
| --- | --- | --- | --- | --- |
|  | Crude | **(No race)** | **(Maternal race only)** | **(Paternal race and only)** |
| Parameter | ORs of TLBW | ORs of TLBW | ORs of TLBW | ORs of TLBW |
|  | β Estimate (95% CI) | β Estimate (95% CI) | β Estimate (95% CI) | β Estimate (95% CI) |
| PM2.5 |  |  |  |  |
| Trimester 1 | 0.96 (0.92, 1.00) | 0.96 (0.92, 0.99) | 0.96 (0.92, 0.99) | 0.96 (0.92, 1.0) |
| Trimester 2 | 0.99 (0.96, 1.03) | 0.99 (0.96, 1.03) | 0.99 (0.96, 1.03) | 0.99 (0.96, 1.03) |
| Trimester 3 | 1.00 (0.97, 1.02) | 1.00 (0.97, 1.02) | 1.00 (0.97, 1.02) | 1.00 (0.97, 1.02) |
| Whole Pregnancy | 1.00 (0.97, 1.03) | 0.99 (0.96, 1.02) | 0.99 (0.96, 1.02) | 0.99 (0.96, 1.03) |

Notes: All analyses are weighted to take the complex sampling design into account. Models adjusted for covariates that were stat sig <0.05 in the non-pollutant models from Supplementary Table 13 (gender, parity, gestation, mom marital, region, tobacco and poverty).

**Supplementary Table 15. Adjusted odds ratios of term low birth weight (LBW) associated with average PM2.5 exposures by trimester and whole pregnancy among the ECLS-B study population stratified by maternal and paternal race**

|  |  |  | |  | |  | |  | | | |  |
| --- | --- | --- | --- | --- | --- | --- | --- | --- | --- | --- | --- | --- |
| Parameter | ORs of TLBW | ORs of TLBW | | ORs of TLBW | | ORs of TLBW | | | ORs of TLBW | ORs of TLBW | | |
|  | β Estimate  (95% CI) | β Estimate  (95% CI) | | | β Estimate  (95% CI) | β Estimate (95% CI) | β Estimate  (95% CI) | | | β Estimate  (95% CI) | | |
|  | **Maternal Race/Ethnicity** | | | | | | | | | | | |
|  | White | | Black | Hispanic | | AAPI^a^ | AI/AN | | |  | | |
| PM2.5 |  |  | |  | |  |  | | |  | | |
| Trimester 1 | 0.99 (0.95, 1.04) | 0.99 (0.92, 1.07) | | 0.88 (0.81, 0.97) | | 0.91 (0.77, 1.08) | 1.05 (0.75, 1.47) | | | |  | |
| Trimester 2 | 0.97 (0.93, 1.02) | 1.05 (0.98, 1.11) | | 0.97 (0.87, 1.07) | | 0.98 (0.91, 1.05) | 0.95 (0.74, 1.21) | | | |  | |
| Trimester 3 | 0.99 (0.96, 1.03) | 1.03 (0.97, 1.09) | | 0.96 (0.90, 1.03) | | 0.95 (0.88, 1.03) | 0.98 (0.76, 1.27) | | |  | | |
| Whole Pregnancy | 0.98 (0.94, 1.02) | 1.04 (0.98, 1.11) | | 0.96 (0.88, 1.04) | | 0.93 (0.84, 1.03) | 1.02 (0.77, 1.36) | | |  | | |
|  | **Paternal Race/Ethnicity** | | | | | | | | | | | |
|  | White | Black | | Hispanic | | AAPI^a^ | AI/AN | | | Not Stated | | |
| PM2.5 |  |  | |  | |  |  | | |  | | |
| Trimester 1 | 0.98 (0.92, 1.04) | 0.96 (0.86, 1.06) | | 0.99 (0.92, 1.07) | | 0.94 (0.87, 1.01) | 0.92 (0.71, 1.19) | | | 1.00 (0.87, 1.15) | | |
| Trimester 2 | 0.95 (0.90, 1.00) | 1.02 (0.95, 1.10) | | 0.88 (0.80, 0.97) | | 0.90 (0.76, 1.07) | 0.74 (0.46, 1.19) | | | 1.07 (0.98, 1.17) | | |
| Trimester 3 | 0.98 (0.94, 1.01) | 0.98 (0.92, 1.04) | | 0.94 (0.86, 1.03) | | 1.01 (0.96, 1.07) | 0.94 (0.82, 1.08) | | | 1.10 (1.03, 1.18) | | |
| Whole Pregnancy | 0.96 (0.91, 1.01) | 0.99 (0.92, 1.07) | | 0.94 (0.88, 1.01) | | 0.98 (0.91, 1.07) | 0.82 (0.66, 1.02) | | | 1.10 (1.00, 1.21) | | |
| Maternal Race/Ethnicity^b^ | | | | | | | | | | | | |
|  | White | Black | | Hispanic | | AAPI^a^ | AI/AN | | |  | | |
| PM2.5 |  |  | |  | |  |  | | |  | | |
| Trimester 1 | 1.00 (0.95, 1.04) | 0.99 (0.93, 1.07) | | 0.88 (0.79, 0.97) | | 0.89 (0.74, 1.07) | 4.08 (1.72, 9.70) | | |  | | |
| Trimester 2 | 0.97 (0.93, 1.02) | 1.04 (0.98, 1.11) | | 0.96 (0.86, 1.06) | | 0.98 (0.90, 1.06) | 0.96 (0.74, 1.25) | | |  | | |
| Trimester 3 | 0.99 (0.96, 1.02) | 1.03 (0.97, 1.09) | | 0.95 (0.89, 1.02) | | 0.95 (0.87, 1.03) | 0.98 (0.75, 1.28) | | |  | | |
| Whole Pregnancy | 0.98 (0.94, 1.02) | 1.04 (0.98, 1.10) | | 0.95 (0.87, 1.03) | | 0.93 (0.84, 1.03) | 1.05 (0.80, 1.37) | | |  | | |

Notes: All analyses are weighted to take the complex sampling design into account. Models adjusted for covariates that were statistically significant <0.05 in the non-pollutant models from Supplementary Table 13 (gender, parity, gestation, mom marital, region, tobacco and poverty). ^a^Asian American and Pacific Islander (AAPI), includes Chinese, Japanese, Hawaiian, Filipino, Asian Indian, Korean, Samoan, Vietnamese, Guamanian and other Asian or Pacific Islander. ^b^Also adjusted for paternal race.
